# Synthesis and Bioactivities of Novel Galactoside Derivatives Containing 1,3,4-Thiadiazole Moiety

**DOI:** 10.3389/fchem.2022.910710

**Published:** 2022-05-19

**Authors:** Yafei Shu, Meihang Chen, Daowang Lu, Zengyan Zhou, Jianhong Yu, Xiaoling Hu, Jiaqin Yang, Aiqin Li, Jianglong Liu, Hairong Luo

**Affiliations:** Colleges of Material and Chemistry Engineering, Tongren University, Tongren, China

**Keywords:** galactoside, thiadiazole, aromatic amide, synthesis, bioactivity

## Abstract

A series of novel galactoside derivatives containing 1,3,4-thiadiazole moiety were synthesized, and the structure of them was verified by spectroscopy of NMR and HRMS, and antifungal and antibacterial activities of them were screened. The results showed that the newly synthesized compounds had good antifungal activities. Among them, Ⅲ16, Ⅲ17, and Ⅲ19 exhibited satisfactory activities against *Phytophthora infestans (P. infestans)*, with EC_50_ values of 5.87, 4.98, and 6.17 μg/ml, respectively, which were similar to those of dimethomorph (5.52 μg/ml). Meanwhile, the title compounds also possessed certain antibacterial activities.

## Introduction

Galactoside and its derivatives are widely found in litchi, laver, seaweed, and snails (Choucry et al., 2021; Kumar, 2020) and had anticancer (Tacke et al., 2017; Oueslati et al., 2020), antiviral (Cai et al., 2006; Abu-Zaied et al., 2021), and antibacterial (Upadhyay et al., 2010) activities. In addition, it was found that novel galactoside derivatives containing a pyrimidine moiety possessed good antifungal activities against *Gibberella zeae* (*G. zeae*), *Botryosphaeria dothidea* (*B. dothidea*), *Phytophthora infestans* (*P. infestans*), *Thanatephorus cucumeris* (*T. cucumeris*), and *Phompsis* sp in the preliminary working of our group (Chen M. H. et al., 2021; Chen et al., 2022)*.* Moreover, it has been reported that glycosylation can improve the properties of active lead compounds, such as solubility, stability, and bioactivity (Wu et al., 2014; Gurung et al., 2017).

It is known that nitrogen-containing heterocyclic compounds have not only a broad spectrum of biological activity and diversity of structure changes but also low toxicity to most warm-blooded animals, birds, fish, and bees (Mermer et al., 2021). 1,3,4-Thiadiazole derivatives, important nitrogen-containing heterocyclic compounds, showed a wide range of bioactivities, such as antifungal (Bhinge et al., 2015; Chudzik et al., 2019), antibacterial (Wu et al., 2021), anticancer (Abas et al., 2021; Avvaru et al., 2021), and antiviral (Yu et al., 2017) activities. In our previous working, 1,3,4-thiadiazole derivatives of glucosides showed good antibacterial and antifungal activities (Chen M. et al., 2021).

**FIGURE 1:**
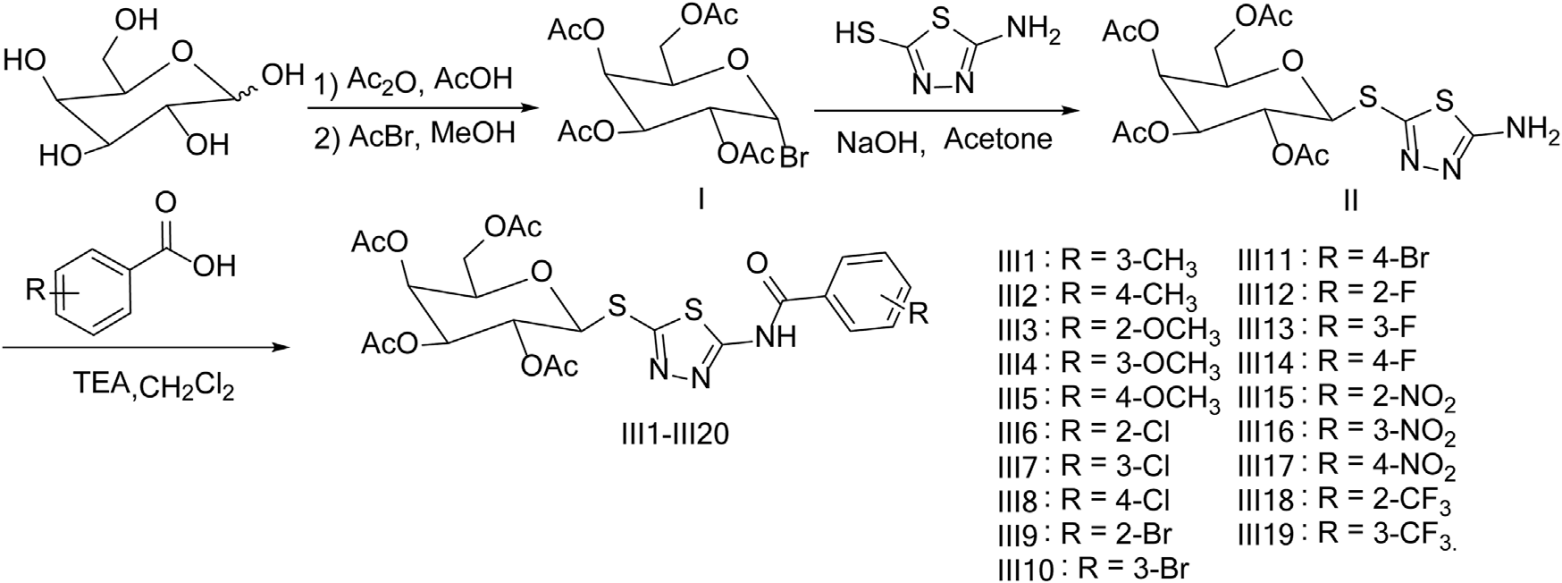
Synthetic route of the target compounds Ⅲ1–Ⅲ19.

In order to find novel structure and effective biological activity of galactoside derivatives, 19 novel galactoside derivatives containing 1,3,4-thiadiazole moiety were synthesized by five reactions and were designed under the guidance of the active substructure splicing method by retaining a part of 1,3,4-thiadiazole and replacing the original glucoside with galactoside on the basis of our previous working (Figure 1). Then, the newly synthesized title compounds are tested for antibacterial and antifungal activities.

## Experimental

### Materials and Instruments

**A**ll solvents and reagents were purchased from commercial suppliers and met the standards. ^1^H NMR and ^13^C NMR spectra were obtained using Bruker DPX 400 MHz and Bruker DPX 600 MHz spectrometers (Bruker, Germany) in DMSO-*d*6 or CDCl3 solution. High-resolution mass spectrometry (HRMS) of the title compounds was performed using an Agilent Technologies mass spectrometer (Agilent Technologies, United States).

### Chemistry

#### General Synthesis Procedures for Intermediate Ⅱ

Intermediate Ⅰ was synthesized by referring to the method of the literature (Kamat et al., 2007; Chen M. et al., 2021). The crude product of intermediate Ⅰ is used directly for the next step of the reaction. To 2-amino-5-mercapto-1,3,4-thiadiazole (1.33 g, 10.0 mmol), 50 ml acetone and 40% sodium hydroxide solution (10 ml) were added successively into a 100-ml two-necked bottle. Then, a solution of intermediate Ⅰ (4.11 g, 10.0 mmol) in acetone (5 ml) was added and maintained under stirring for about 30 min (Scattolin et al., 2020; Chen M. et al., 2021). After the reaction was completed, the mixture was concentrated, and 30 ml of water was added and extracted with dichloromethane (3 × 20 ml), and the organic layer was concentrated and recrystallized with ethyl acetate to afford the intermediate Ⅱ (3.9 g, yield: 84%) as a white solid. ^1^H NMR (600 MHz, DMSO-*d6*) δ 7.48 (s, 2H, NH_2_), 5.33 (s, 1H, H-1´), 5.30–5.21 (m, 2H, H-2´, H-3´), 5.05 (t, *J* = 9.9 Hz, 1H, H-4´), 4.33 (t, *J* = 6.2 Hz, 1H, H-5´), 4.13–4.00 (m, 2H, H-6´, H-6´´), 2.14 (s, 3H, CH_3_), 2.07 (s, 3H, CH_3_), 2.02 (s, 3H, CH_3_), and 1.93 (s, 3H, CH_3_).

#### General Synthesis Procedures for Title Compounds Ⅲ1-Ⅲ19

Substituted benzoic acid (2.4 mmol) was added to 4 ml thionyl chloride in batches with magnetic stirring and refluxed for 2.0 h (monitored by TLC). The solvent was removed under negative pressure; dichloromethane (2 ml) was added into the residue to give a light yellow solution, which was added dropwise into a mixture of the intermediate Ⅱ (0.93 g, 2.0 mmol), 15 ml dichloromethane, and triethylamine (0.24 g, 2.4 mmol) (Chen M. et al., 2021). After the reaction was completed, 10 ml water was added into the mixture and divided, and the organic layer was concentrated to the crude product. The crude product was recrystallized with isopropanol to afford the title compounds Ⅲ1–Ⅲ19. The characterization details of the title compounds Ⅲ2–Ⅲ19 are presented in the Supplemental Material.

(2*R*,3*S*,4*S*,5*R*,6*S*)-2-(acetoxymethyl)-6-((5-(3-methylbenzamido)-1,3,4-thiadiazol-2-l)thio)tetrahydro-2*H*-pyran-3,4,5-triyltriacetate (Ⅲ1): white solid, yield 75.0%, m.p. 159–161°C; 1H NMR (400 MHz, CDCl_3_) *δ* 12.33 (s, 1H, NH), 8.01 (s, 1H, Ar-H), 7.95 (d, *J* = 7.3 Hz, 1H, Ar-H), 7.55–7.37 (m, 2H, Ar-H), 5.48 (d, *J* = 2.9 Hz, 1H, H-3´), 5.38 (t, *J* = 10.0 Hz, 1H, H-1´), 5.11 (dd, *J* = 10.0, 3.3 Hz, 1H, H-2´), 5.03 (d, *J* = 10.1 Hz, 1H, H-4´), 4.20 (d, *J* = 7.6 Hz, 2H, H-5´, H-6´), 3.99 (t, *J* = 6.4 Hz, 1H, H-6´´), 2.48 (s, 3H, CH_3_), 2.20 (s, 3H, CH_3_), 2.10 (s, 6H, 2×CH_3_), and 2.00 (s, 3H, CH_3_). ^13^C NMR (150 MHz, CDCl_3_) *δ* 172.48, 170.58, 170.45, 169.89, 169.80, 154.72, 138.62, 134.24, 131.59, 129.35, 129.11, 126.02, 83.10, 74.70, 71.16, 68.06, 67.32, 62.39, 40.41, 40.27, 40.13, 39.99, 39.85, 39.71, 39.57, 21.35, 20.90, and 20.78; HRMS [M+H]^+^ calculated for C_24_H_27_N_3_O_10_S_2_: m/z 582.1216, found 582.1210.

### Antifungal Activity *In Vitro*

The antifungal activity of the title compounds Ⅲ1–Ⅲ19 against *G. zeae, B. dothidea, Phompsis* sp., *P. infestans,* and *T. cucumeris in vitro* were tested by a mycelia growth method at 50 μg/ml (Maddila et al., 2016; Chen M. H. et al., 2021; Chen M. et al., 2021; Chen et al., 2022). Dimethomorph was used as a positive control, and DMSO was used as a negative control, and each treatment was operated in three replicates. Subsequently, the title compounds Ⅲ16, Ⅲ17, and Ⅲ19 were further evaluated for their corresponding antifungal EC_50_ values with three replicates and used dimethomorph as the positive controls.

### Antibacterial Activity *In Vitro*

The antibacterial activity of the title compounds Ⅲ1–Ⅲ19 against *Xcc* and *Xoo in vitro* was tested using the turbidimeter test at 200 and 100 μg/ml (Dalgaard et al., 1994; Yu et al., 2017; Chen M. H. et al., 2021; Chen et al., 2022). Thiodiazole-copper was used as a positive control, and DMF was used as a negative control, and each treatment was operated in three replicates.

## Result and Discussion

### Synthesis

The method of the synthesis for title compounds was listed as follows: Intermediate Ⅰ was synthesized by galactose acetylation and bromination, and then intermediate Ⅰ reacted with 2-amino-5-mercapto-1,3,4-thiadiazole to give intermediate Ⅱ; substituted benzoic acids were chlorinated by thionyl chloride and reacted with intermediate Ⅱ to produce the title compounds Ⅲ1–Ⅲ19. Moreover, to optimize the reaction conditions of the key intermediate Ⅱ, the influence of catalyst, temperature, and solvent were tested and are listed in Table 1. The results indicated that the catalyst, solvent, and temperature had a pronounced effect on the yield, and a maximum yield of 82% was achieved when sodium hydroxide was used as a catalyst and acetone as a solvent for 0.5 h at room temperature.

**TABLE 1:** Reaction conditions for intermediate Ⅱ were optimized.

Entry	Catalyst	Solvent	Temperature/°C	Yield^a^ (%)
1	NaHCO_3_	CH_2_Cl_2_	r.m.	18
2	Na_2_CO_3_	CH_2_Cl_2_	r.m.	30
3	NaOH	CH_2_Cl_2_	r.m.	72
3	Et_3_N	CH_2_Cl_2_	r.m.	55
4	NaOH	THF	r.m.	66
5	NaOH	CHCl_3_	r.m.	70
6	NaOH	CH_3_CN	r.m.	66
7	NaOH	(CH_3_)_2_CO	r.m.	82
8	NaOH	(CH_3_)_2_CO	0°C	72
9	NaOH	(CH_3_)_2_CO	50°C	78
10	NaOH	(CH_3_)_2_CO	Reflux	81

### Antifungal Activity *In Vitro*

The antifungal activity of the title compounds Ⅲ1–Ⅲ19 against *G. zeae, B. dothidea, P. infestans, Phompsis* sp., and *T. cucumeris* are listed in Table 2. Table 2 indicated that Ⅲ1–Ⅲ19 showed good antifungal activities, with the inhibition rates of 21.5%–63.4%, 21.6%–66.0%, 23.6%–80.1%, 32.5%–58.1%, and 33.4–68.4% at 50 μg/ml, respectively. Among them, Ⅲ16, Ⅲ17, and Ⅲ19 exhibited satisfactory *in vitro* antifungal activities against *P. infestans*, with the inhibition rates of 80.1, 79.7, and 79.3%, respectively, which were equal to those of dimethomorph (78.2%). Based on the aforementioned results, the EC_50_ values of Ⅲ16, Ⅲ17, and Ⅲ19 were tested and are shown in Table 3. Table 3 indicated that Ⅲ16, Ⅲ17, and Ⅲ19 showed good antifungal activities against *P. infestans,* with EC_50_ values of 5.87, 4.98, and 6.17 μg/ml, respectively, which were similar to those of dimethomorph (5.52 μg/ml) (Chen et al., 2022), and which were comparable to those of the previously found inhibitory activity of glucosides derivatives containing 4-fluorobenzamido-1,3,4-thiadiazole against *P. infestans* (3.43 μg/ml) (Chen M. et al., 2021).

**TABLE 2:** Antifungal activity of compounds Ⅲ1–Ⅲ19 *in vitro* (50 μg/ml).

Compound	Inhibition rate (%)				
	*G. zeae*	*B. dothidea*	*P. infestans*	*Phompsis* sp.	*T. cucumeris*
Ⅲ1	28.8 ± 1.3	24.5 ± 2.0	23.6 ± 2.8	49.2 ± 2.6	45.2 ± 1.7
Ⅲ2	34.5 ± 1.6	32.0 ± 1.0	28.1 ± 1.7	36.3 ± 3.4	33.4 ± 1.4
Ⅲ3	37.6 ± 2.0	31.0 ± 1.8	25.6 ± 2.0	32.5 ± 1.5	56.3 ± 2.1
Ⅲ4	45.4 ± 2.6	25.8 ± 1.2	24.7 ± 1.5	36.2 ± 1.8	42.6 ± 1.7
Ⅲ5	40.1 ± 2.1	26.8 ± 2.6	25.3 ± 2.6	47.5 ± 1.9	47.5 ± 1.8
Ⅲ6	36.2 ± 1.1	21.6 ± 2.8	56.4 ± 1.4	34.2 ± 2.1	43.4 ± 1.5
Ⅲ7	47.0 ± 1.3	33.2 ± 2.3	56.7 ± 3.2	55.6 ± 1.4	46.3 ± 1.5
Ⅲ8	34.2 ± 1.6	48.5 ± 2.1	56.1 ± 1.2	35.2 ± 2.4	35.7 ± 2.4
Ⅲ9	38.6 ± 1.5	54.8 ± 1.7	59.8 ± 2.1	33.5 ± 2.2	45.6 ± 1.8
Ⅲ10	43.0 ± 1.3	51.6 ± 2.0	57.5 ± 3.0	37.3 ± 2.3	55.4 ± 1.4
Ⅲ11	45.4 ± 0.8	50.7 ± 1.2	57.6 ± 2.7	45.1 ± 1.9	43.0 ± 1.2
Ⅲ12	63.4 ± 1.0	50.5 ± 2.3	73.5 ± 2.1	34.5 ± 2.1	59.7 ± 2.2
Ⅲ13	53.8 ± 1.2	46.4 ± 1.6	73.1 ± 1.6	48.1 ± 1.3	68.3 ± 1.8
Ⅲ14	52.3 ± 1.8	66.4 ± 1.2	77.5 ± 2.1	42.6 ± 1.2	56.5 ± 2.1
Ⅲ15	61.0 ± 2.4	65.3 ± 2.6	75.1 ± 2.2	45.2 ± 1.5	56.3 ± 1.3
Ⅲ16	52.2 ± 2.1	66.0 ± 2.5	80.1 ± 1.3	58.1 ± 1.4	56.5 ± 1.6
Ⅲ17	45.2 ± 1.6	54.3 ± 2.4	79.7 ± 1.2	43.2 ± 1.5	58.7 ± 1.0
Ⅲ18	55.4 ± 2.0	55.2 ± 2.0	78.0 ± 2.3	44.5 ± 2.2	65.3 ± 2.0
Ⅲ19	57.2 ± 1.6	54.7 ± 2.5	79.3 ± 2.1	48.2 ± 1.4	68.4 ± 1.9
Dimethomorph	74.3 ± 2.0^a^	72.3 ± 1.6^a^	78.2 ± 1.1^a^	69.3 ± 1.6^a^	68.3 ± 1.6^a^

a Refer to the previous articles of our group (Chen M. et al., 2021).

**TABLE 3:** EC_50_ value of antifungal activity for part of compounds against *P. infestans.*

Compound	Toxic regression equation	*R*	EC_50_ (μg/ml)
Ⅲ16	y = 0.63x + 4.51	0.96	5.87 ± 1.5
Ⅲ17	y = 0.61x + 4.57	0.99	4.98 ± 2.1
Ⅲ19	y = 0.67x + 4.47	0.98	6.17 ± 1.8
Dimethomorph	y = 0.94x + 4.30	0.99	5.52 ± 1.2^a^

a Refer to the previous articles of our group (Chen M. et al., 2021).

### Antibacterial Activity *In Vivo*

Moreover, the antibacterial activities of the title compounds against *Xcc* and *Xoo* were tested at 200 and 100 μg/ml and are listed in Table 4. Table 4 indicated that the title compounds Ⅲ1–Ⅲ19 exhibited certain antibacterial activities against *Xoo* and *Xcc* at 200 and 100 μg/ml, with the inhibition rates of 31.5%–64.2% and 40.8%–57.7% and 18.3%–36.2% and 19.8%–36.1%, respectively, which were lower than those of thiodiazole-copper (70.1, 43.6, and 46.1%), and which were comparable to that of the previously found novel glucoside derivatives containing 1,3,4-thiadiazole moiety with antibacterial activity (Chen M. et al., 2021). Based on the aforementioned results, it was demonstrated that the antifungal and antibacterial activities of compounds replacing the original glucoside with galactoside did not show any improvement, that is, the configuration of the third on the six-member sugar ring has little influence on the antifungal and antibacterial activities.

**TABLE 4:** Antibacterial activity of compounds (Ⅲ1–Ⅲ19) *in vitro*.

Compound	*Xoo*		*Xcc*	
	200 μg/ml	100 μg/ml	200 μg/ml	100 μg/ml
Ⅲ1	47.6 ± 2.5	29.1 ± 1.6	45.4 ± 1.4	28.5 ± 1.2
Ⅲ2	43.5 ± 1.2	23.1 ± 1.3	42.2 ± 2.3	29.2 ± 1.5
Ⅲ3	46.7 ± 1.2	25.4 ± 2.0	44.3 ± 2.1	24.1 ± 1.3
Ⅲ4	45.5 ± 1.1	23.1 ± 1.2	48.0 ± 2.3	29.2 ± 1.4
Ⅲ5	35.3 ± 2.0	18.3 ± 2.4	42.1 ± 1.5	24.7 ± 2.1
Ⅲ6	41.5 ± 2.3	21.5 ± 3.1	45.1 ± 2.1	24.8 ± 1.4
Ⅲ7	36.2 ± 2.8	19.2 ± 3.0	57.7 ± 1.3	36.1 ± 1.7
Ⅲ8	45.4 ± 2.3	26.5 ± 2.1	53.5 ± 2.1	34.8 ± 2.5
Ⅲ9	37.3 ± 1.8	19.1 ± 1.0	55.0 ± 1.8	27.0 ± 1.4
Ⅲ10	43.4 ± 2.6	24.3 ± 1.2	53.1 ± 1.4	26.2 ± 1.2
Ⅲ11	44.2 ± 1.5	27.5 ± 2.7	40.0 ± 1.7	19.8 ± 2.0
Ⅲ12	52.6 ± 2.4	26.8 ± 1.8	41.2 ± 1.0	20.4 ± 1.4
Ⅲ13	56.2 ± 1.1	26.5 ± 3.1	48.1 ± 2.5	25.7 ± 2.5
Ⅲ14	57.6 ± 2.0	29.0 ± 1.0	45.2 ± 1.1	21.2 ± 1.6
Ⅲ15	64.2 ± 1.2	30.3 ± 1.4	54.1 ± 2.9	26.0 ± 1.7
Ⅲ16	58.6 ± 1.2	23.2 ± 2.1	55.1 ± 1.8	27.8 ± 1.1
Ⅲ17	62.8 ± 1.1	34.5 ± 0.9	57.0 ± 2.2	28.9 ± 2.0
Ⅲ18	54.2 ± 1.2	33.0 ± 1.3	49.0 ± 1.0	29.4 ± 2.7
Ⅲ19	53.0 ± 1.4	36.2 ± 2.2	45.4 ± 2.6	23.8 ± 2.5
Thiodiazole-copper	70.1 ± 2.3^a^	43.6 ± 1.5^a^	80.2 ± 1.5^a^	46.1 ± 1.3^a^

a Refer to the previous articles of our group (Chen et al., 2022).

## Conclusion

A total of 19 novel galactoside derivatives containing 1,3,4-thiadiazole moiety were designed under the guidance of the active substructure splicing method and synthesized by five reactions. The bioactivity results indicated that the title compounds exhibited good antibacterial and antifungal activities, while some of them showed excellent antifungal activities. Therefore, it was demonstrated that the galactoside derivatives containing 1,3,4-thiadiazole moiety can be used to develop potential agrochemicals in the future.

## Data Availability

The original contributions presented in the study are included in the article/Supplementary Material, further inquiries can be directed to the corresponding author.
