# Supplementary material for: Synthesis and Bioactivities of Novel Galactoside Derivatives Containing 1,3,4-Thiadiazole Moiety
Source: Front Chem. 2022 May 19;10:910710. doi: 10.3389/fchem.2022.910710 (PMC9160659; doi:10.3389/fchem.2022.910710)
Supplement: Supplementary file 1 [file DataSheet1.pdf]

Supplementary Material

1. Figures of ^{1}H NMR spectrum of the intermediate II

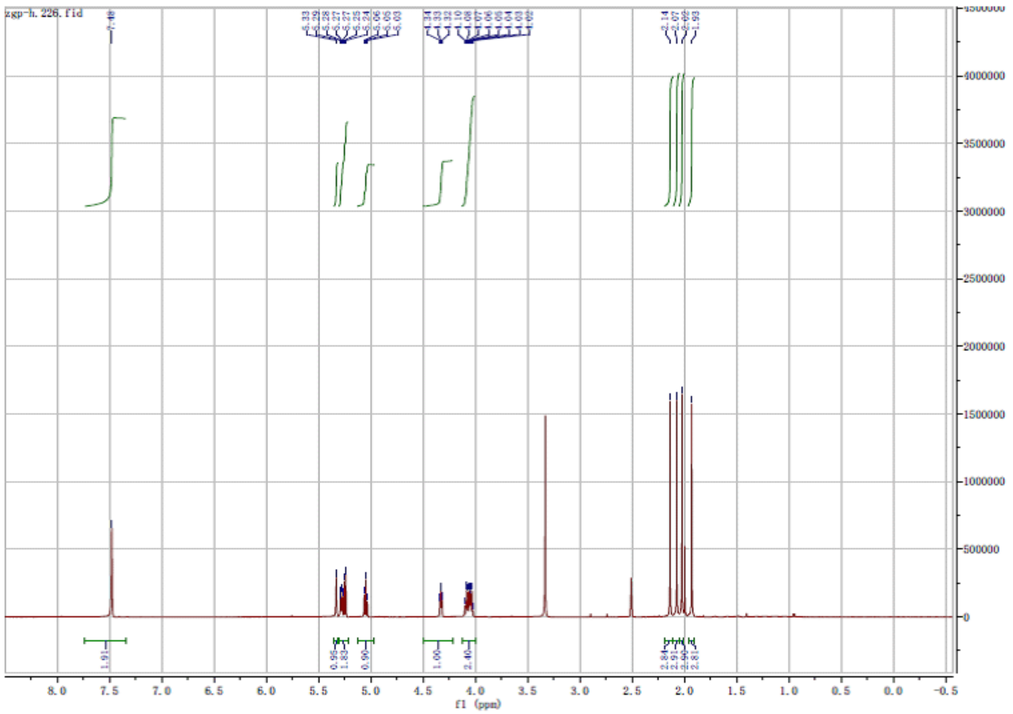

^{1}H NMR spectrum of the intermediate **II**

2. Figures of ^{1}H NMR, ^{13}C NMR and HRMS spectrum of the target compounds (III1-III20)

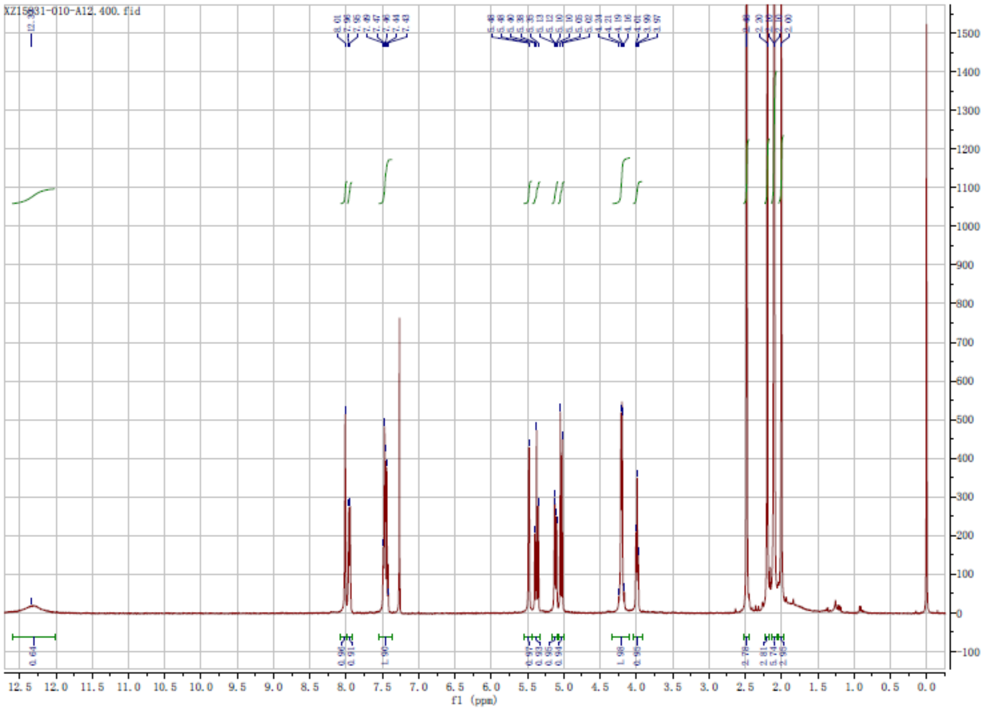

^{1}H NMR spectrum of the target compounds **III1**

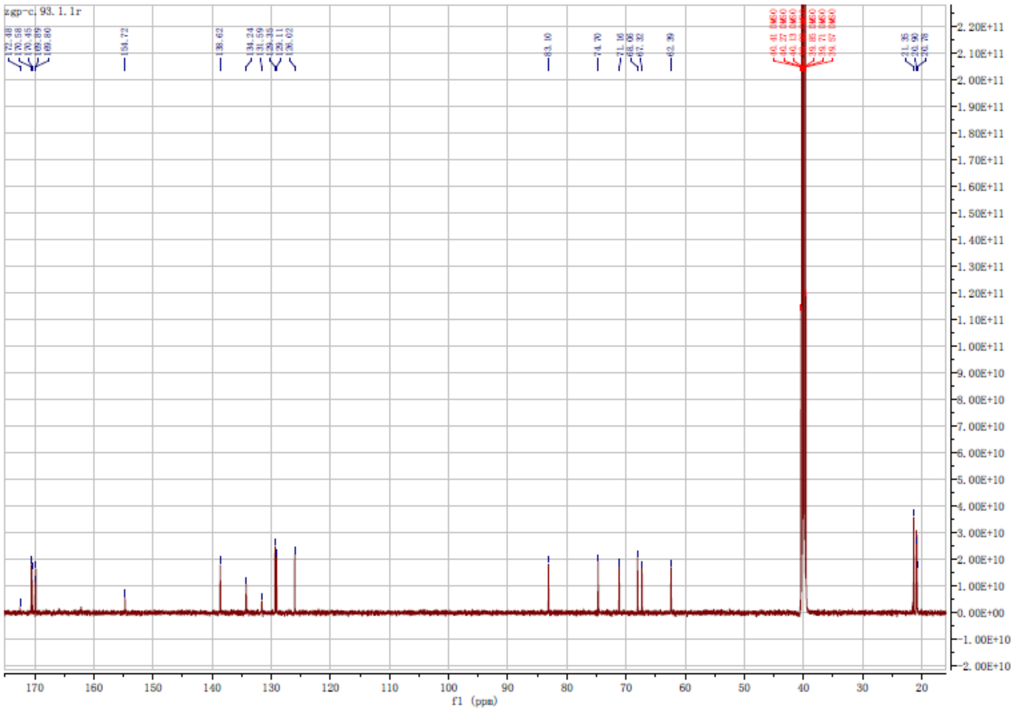

^{13}C NMR spectrum of the target compounds **III1**

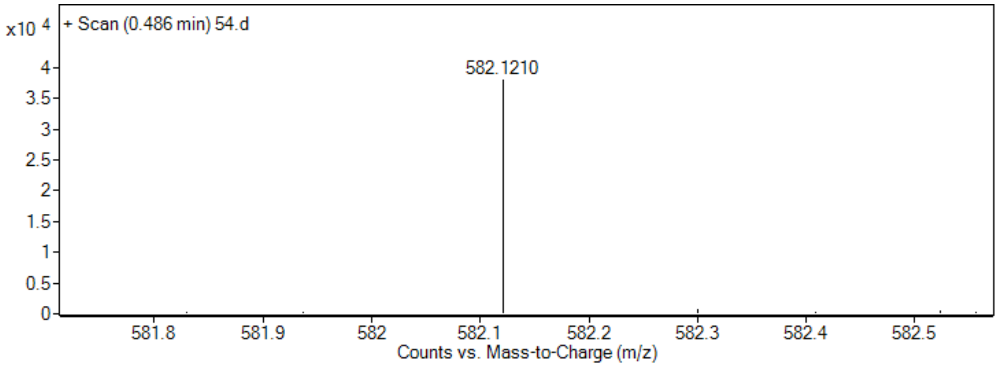

HRMS spectrum of the target compounds **III1**

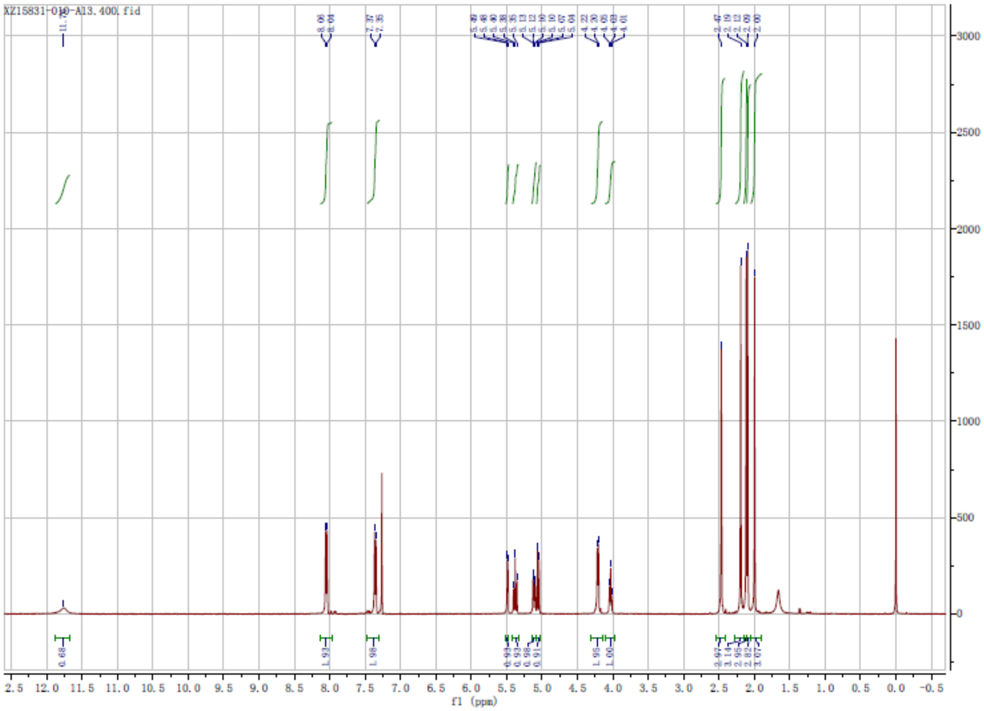

^{1}H NMR spectrum of the target compounds **III2**

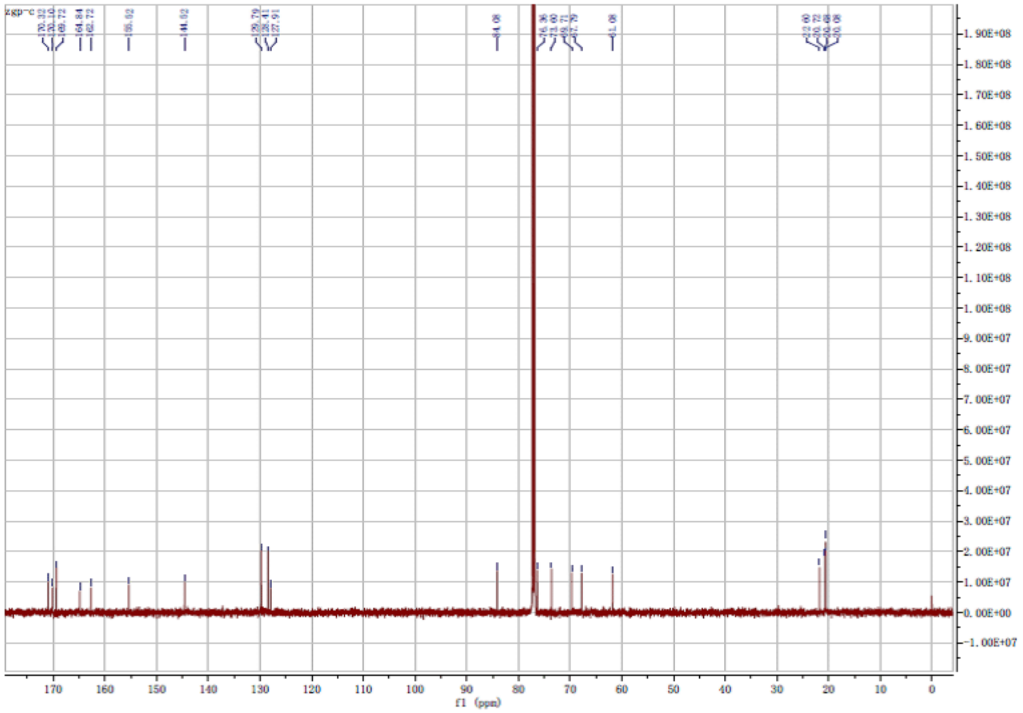

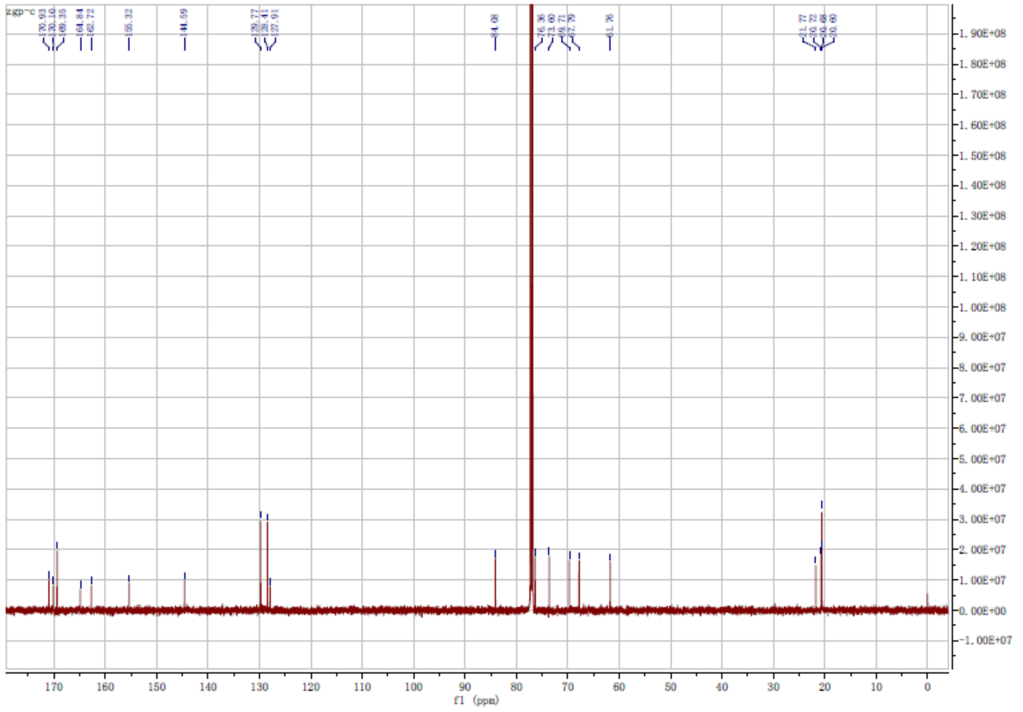

^{13}C NMR spectrum of the target compounds **III2**

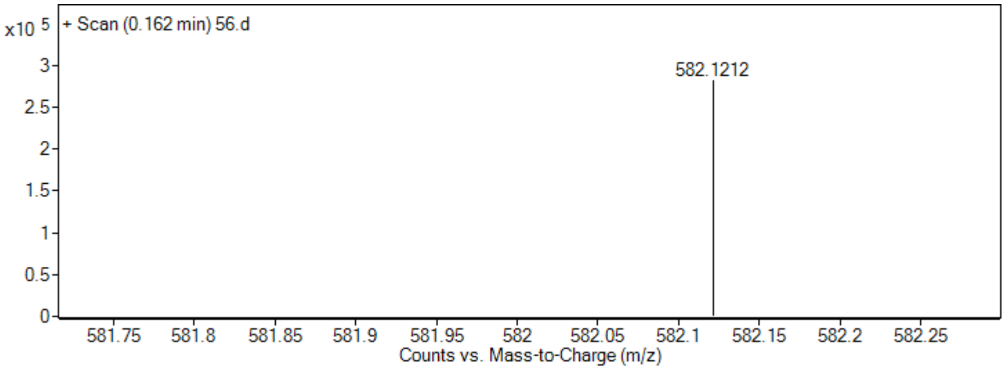

HRMS spectrum of the target compounds **III2**

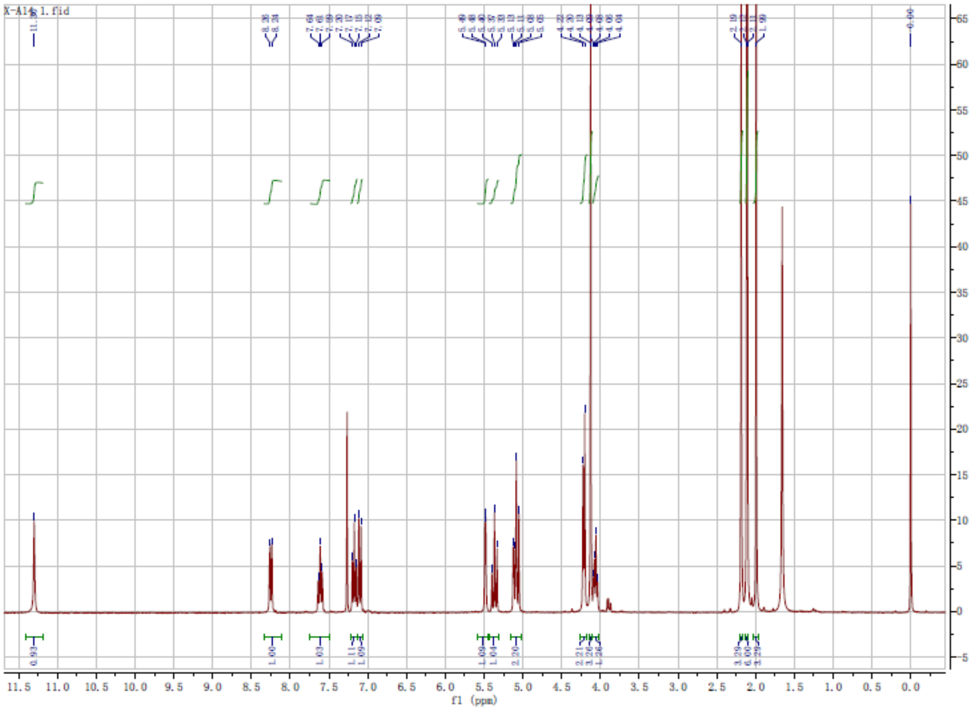

^{1}H NMR spectrum of the target compounds **III3**

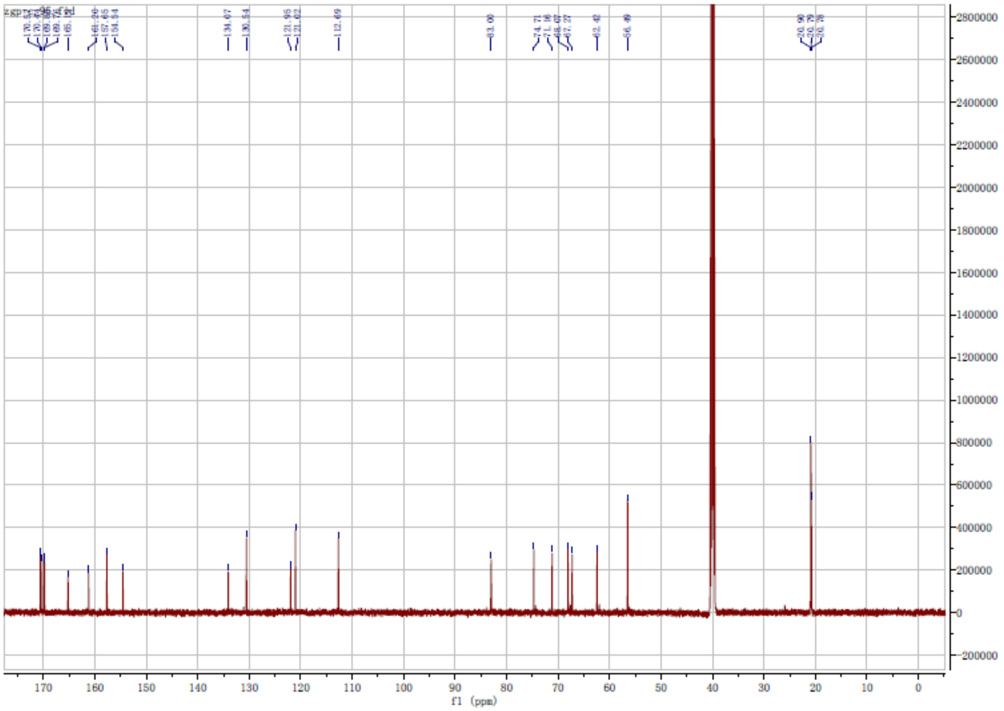

^{13}C NMR spectrum of the target compounds **III3**

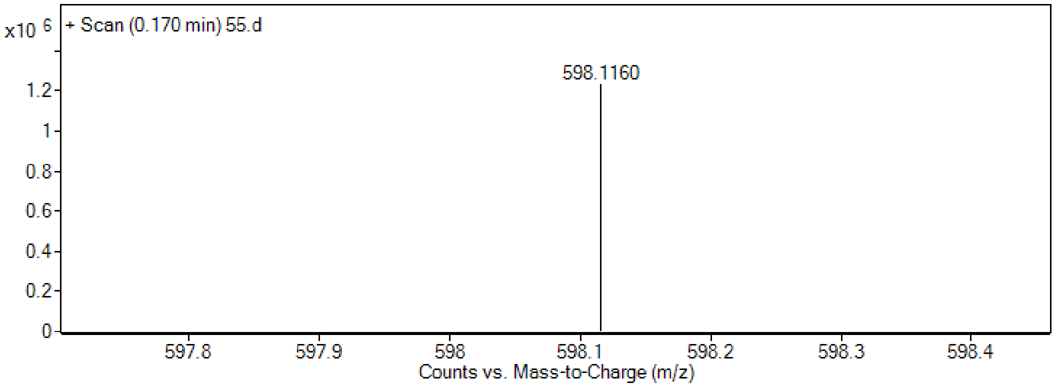

HRMS spectrum of the target compounds **III3**

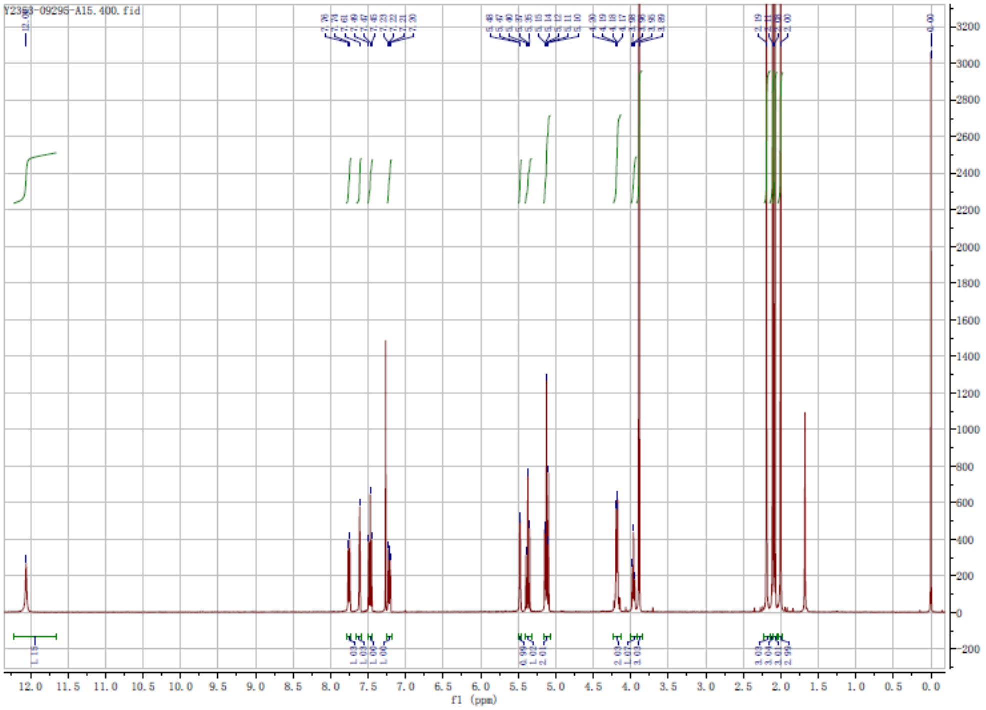

^{1}H NMR spectrum of the target compounds **III4**

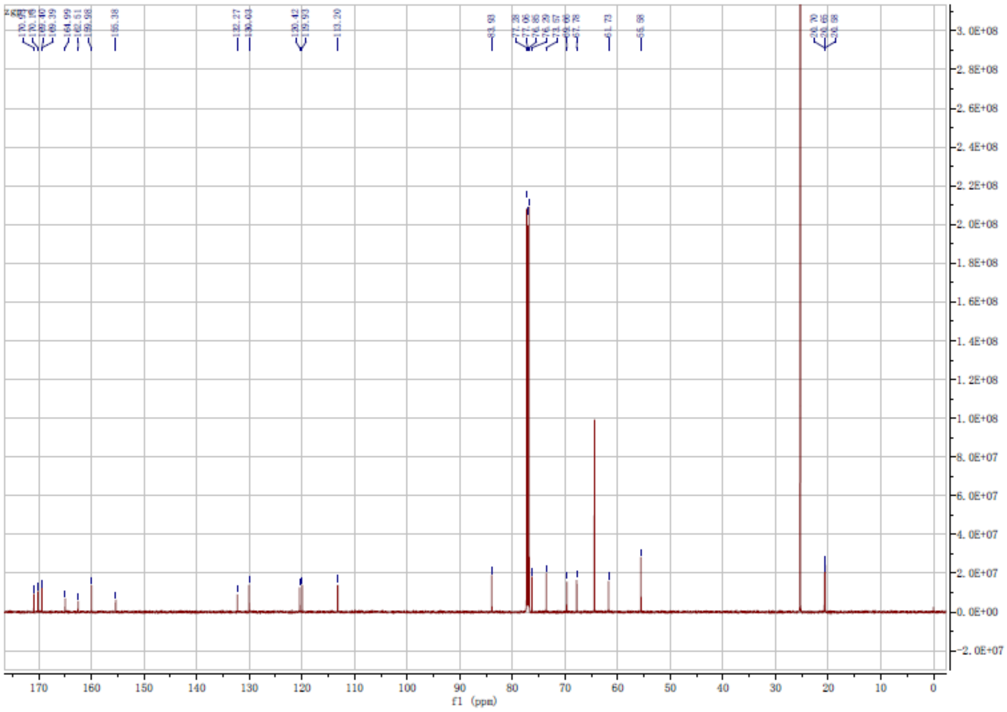

^{13}C NMR spectrum of the target compounds **III4**

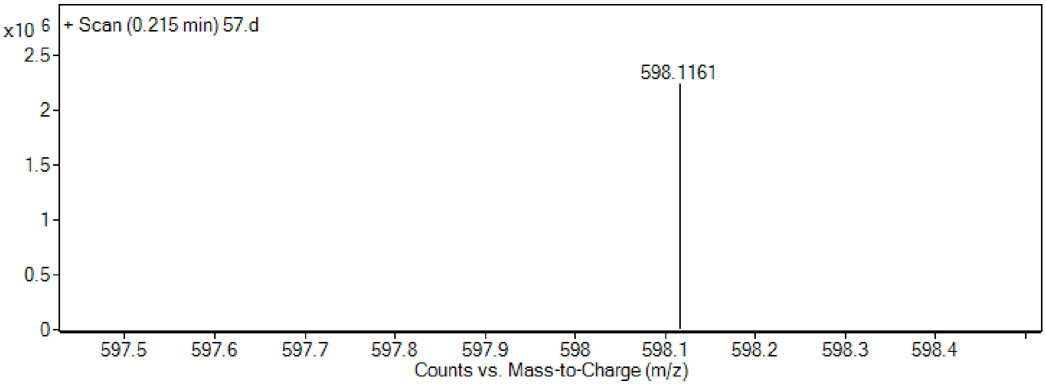

HRMS spectrum of the target compounds **III4**

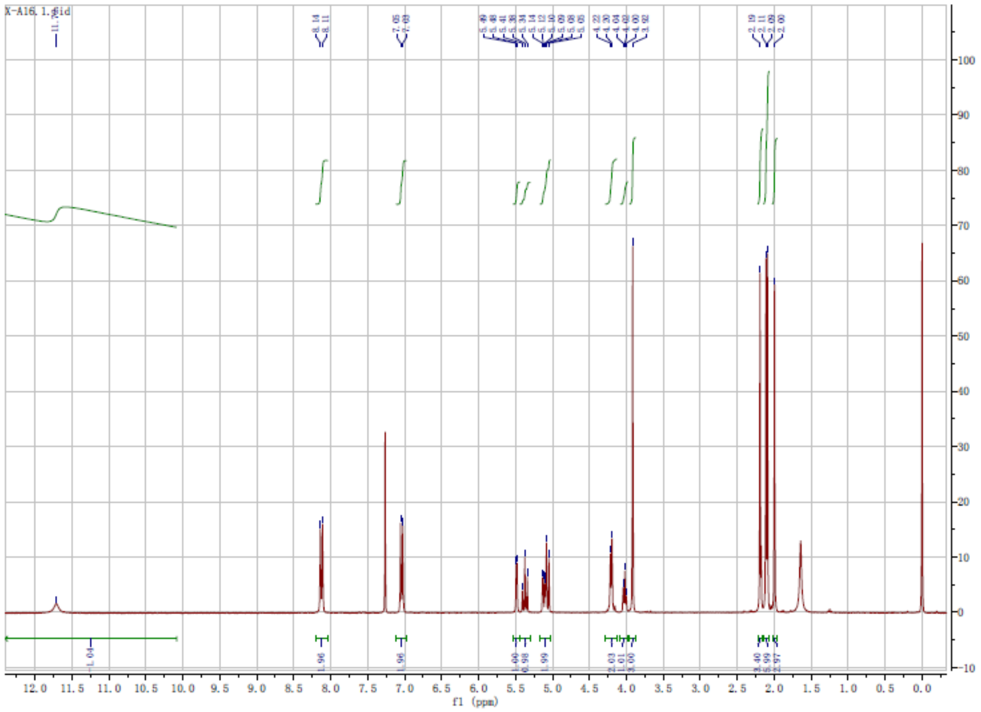

^{1}H NMR spectrum of the target compounds **III5**

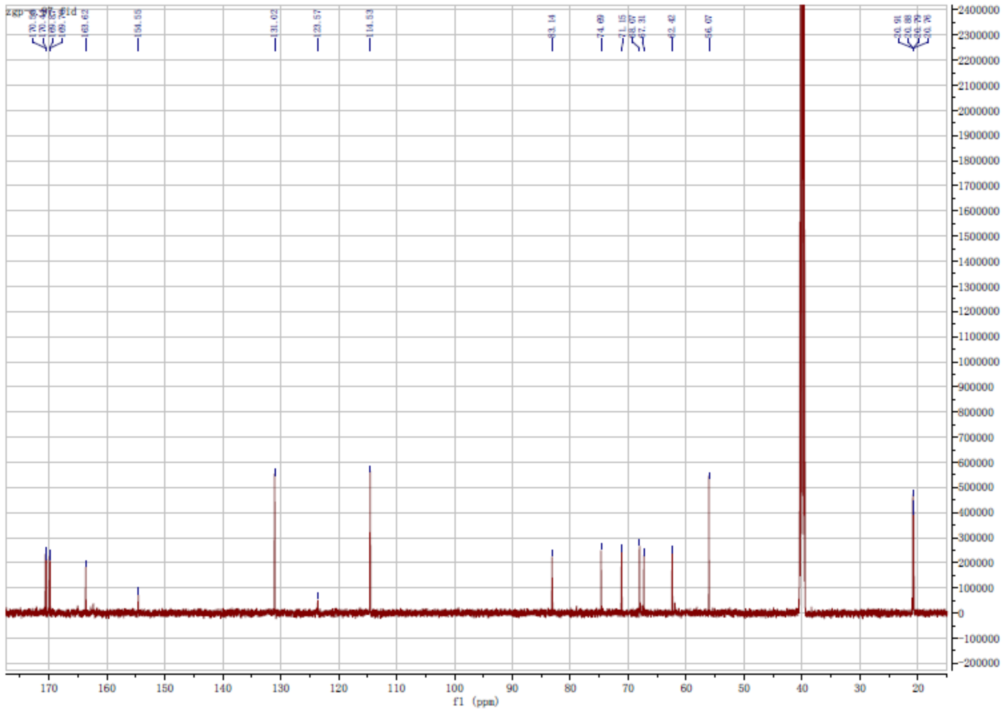

^{13}C NMR spectrum of the target compounds **III5**

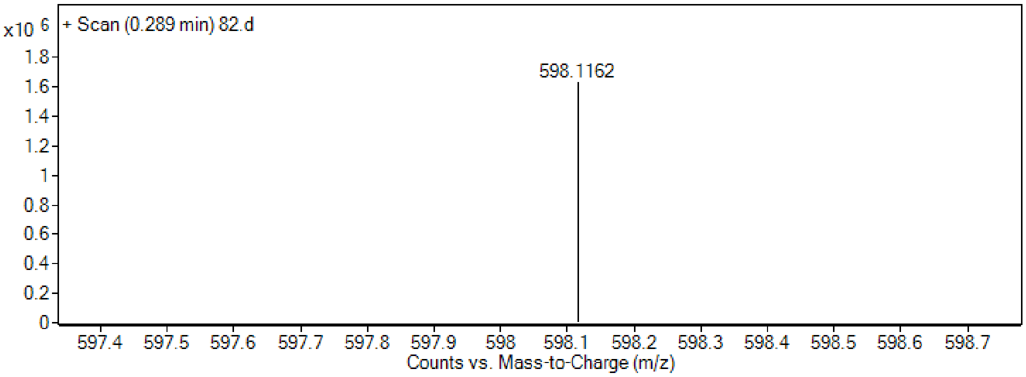

HRMS spectrum of the target compounds **III5**

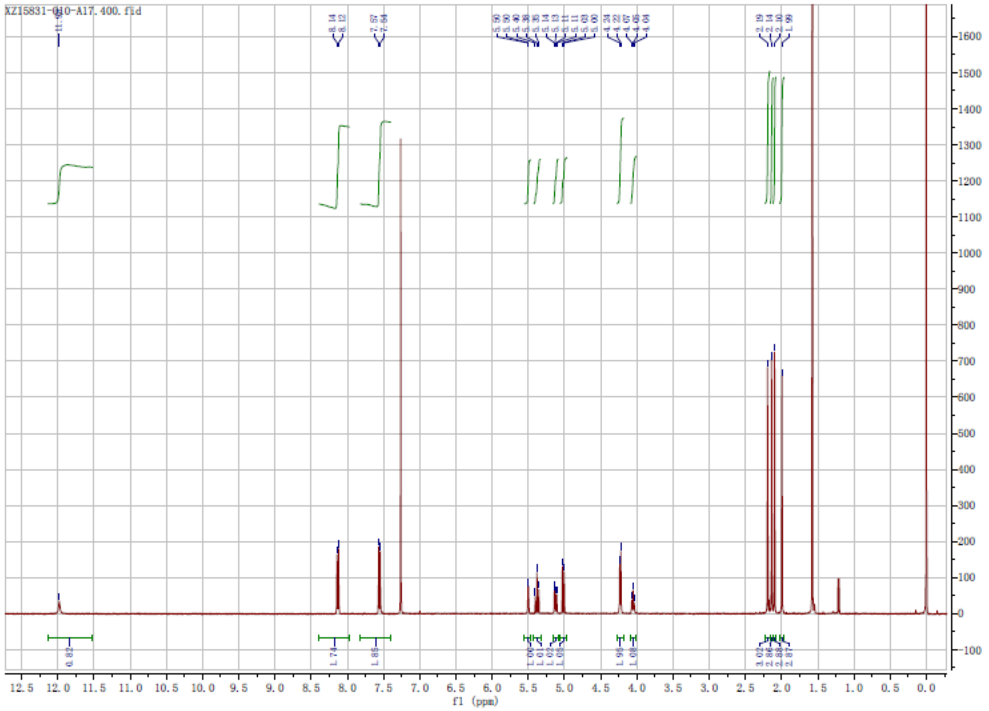

^{1}H NMR spectrum of the target compounds **III6**

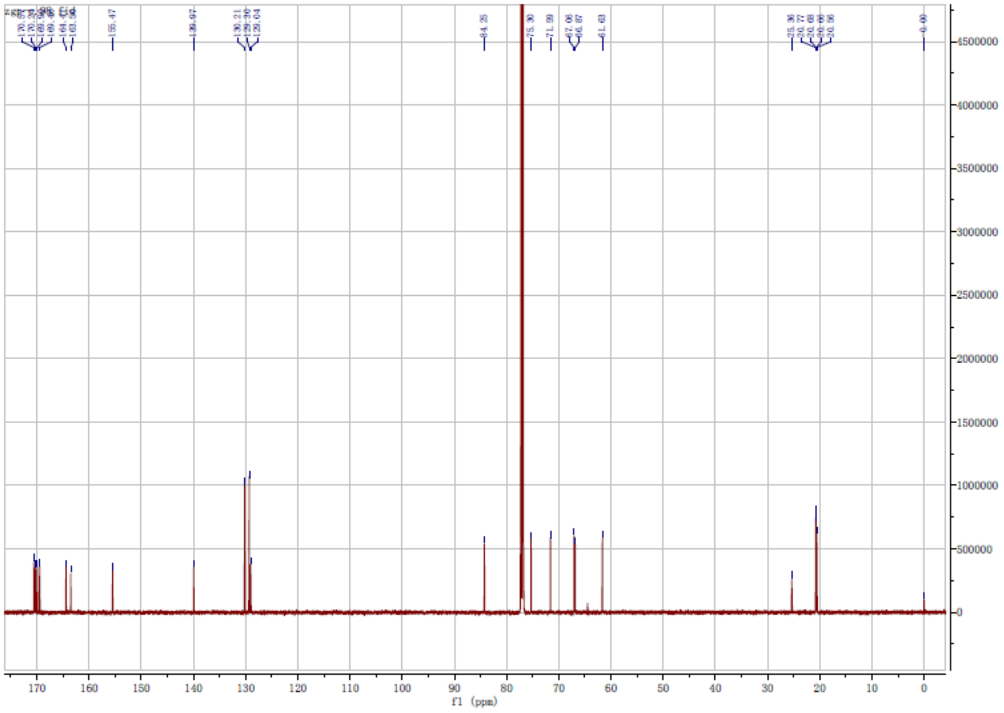

^{13}C NMR spectrum of the target compounds **III6**

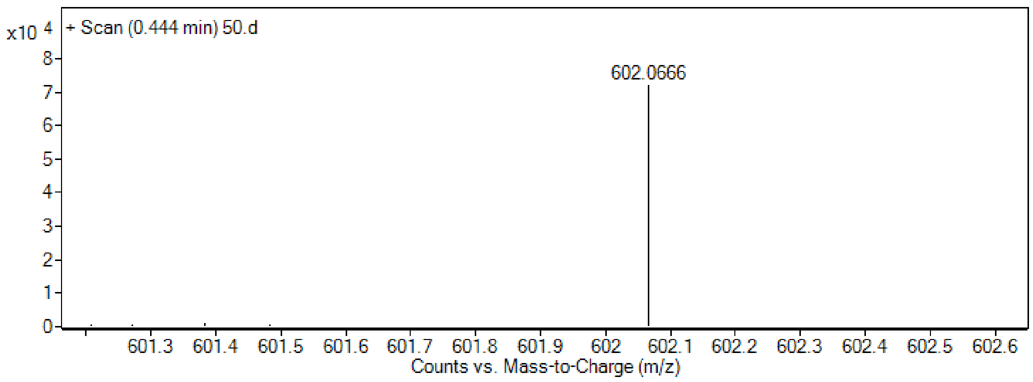

HRMS spectrum of the target compounds **III6**

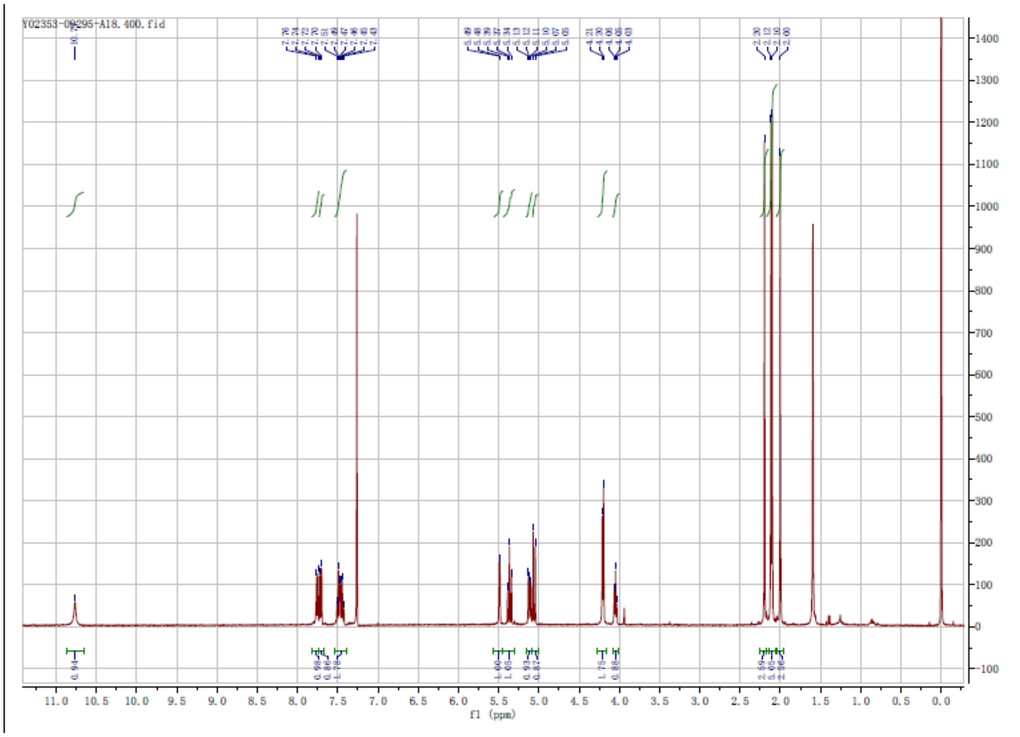

^{1}H NMR spectrum of the target compounds **III7**

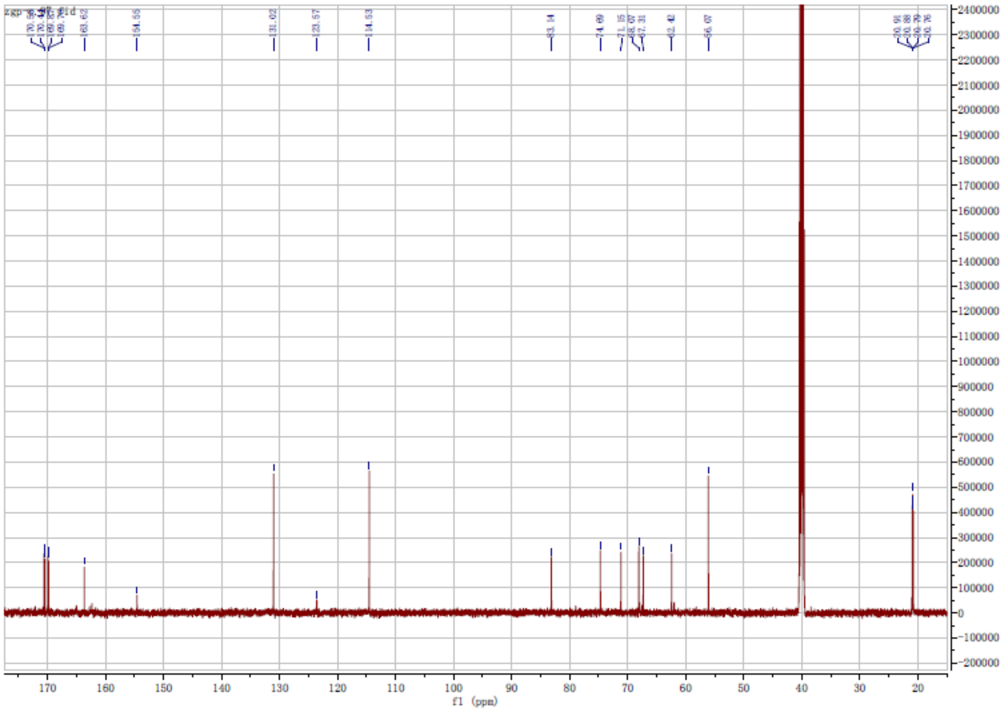

^{13}C NMR spectrum of the target compounds **III7**

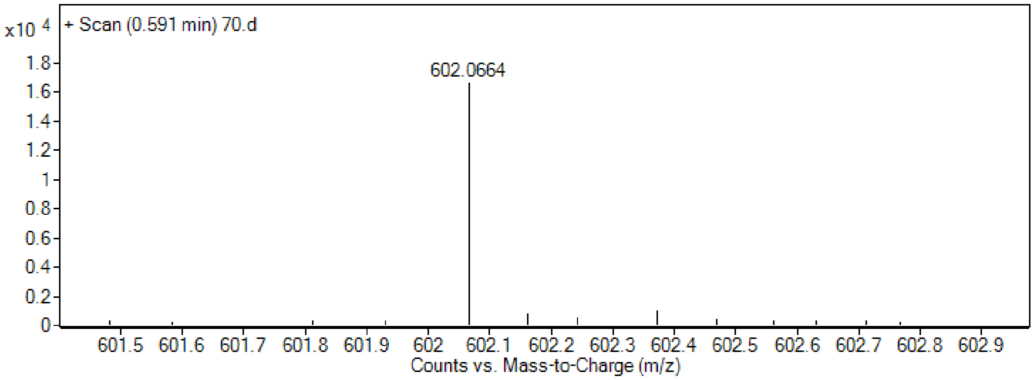

HRMS spectrum of the target compounds **III7**

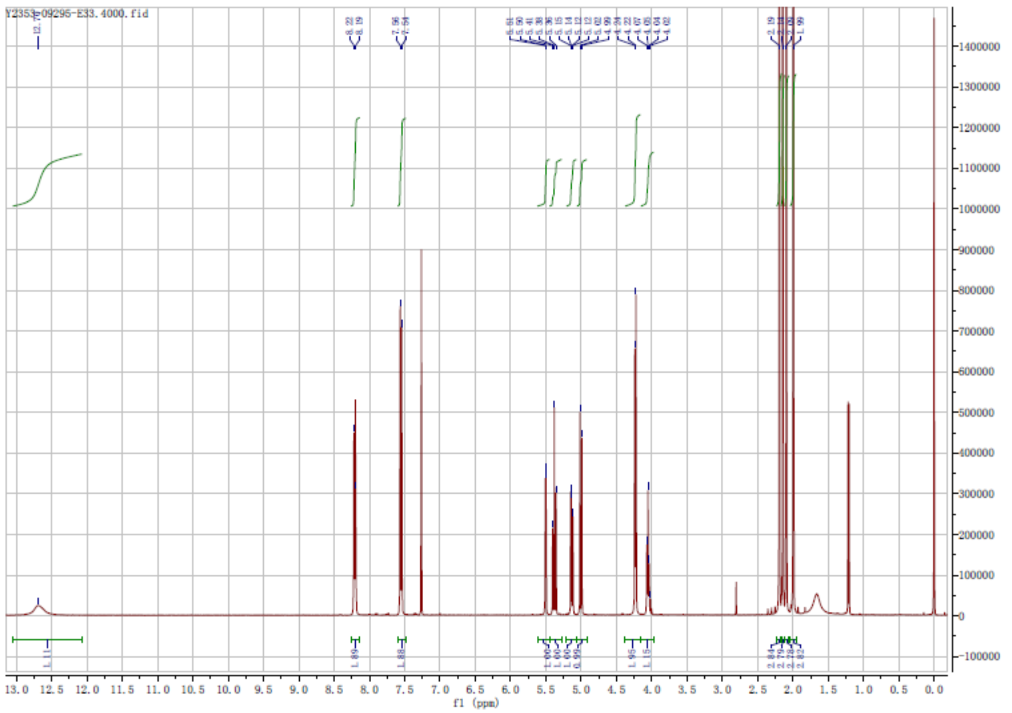

^{1}H NMR spectrum of the target compounds **III8**

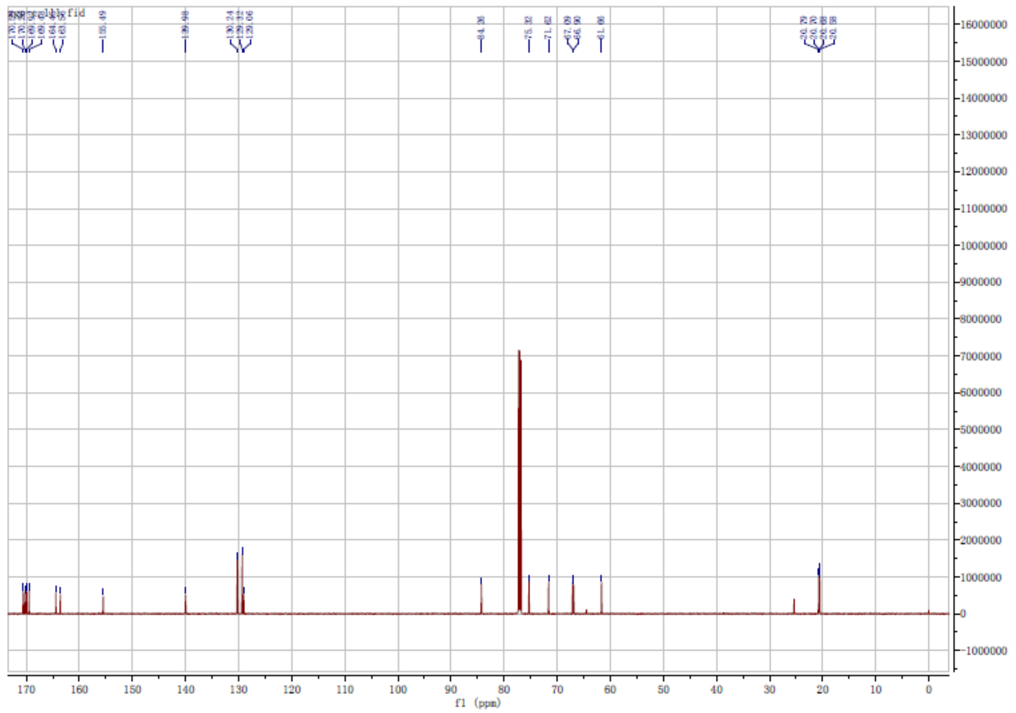

^{13}C NMR spectrum of the target compounds **III8**

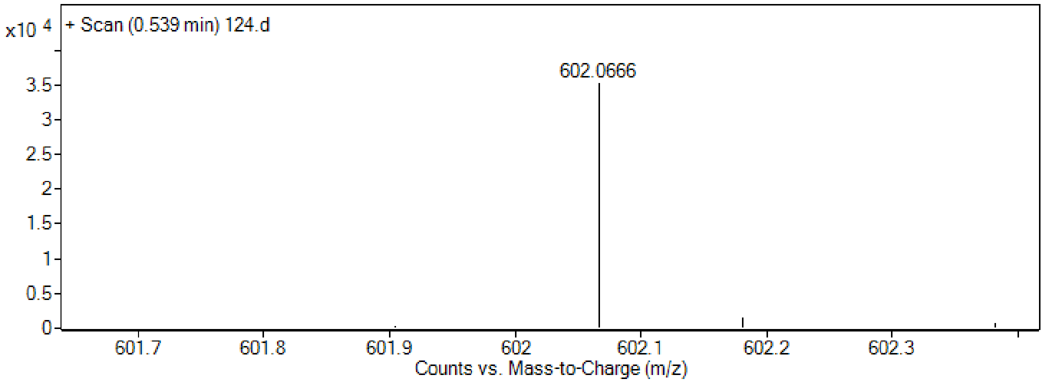

HRMS spectrum of the target compounds **III8**

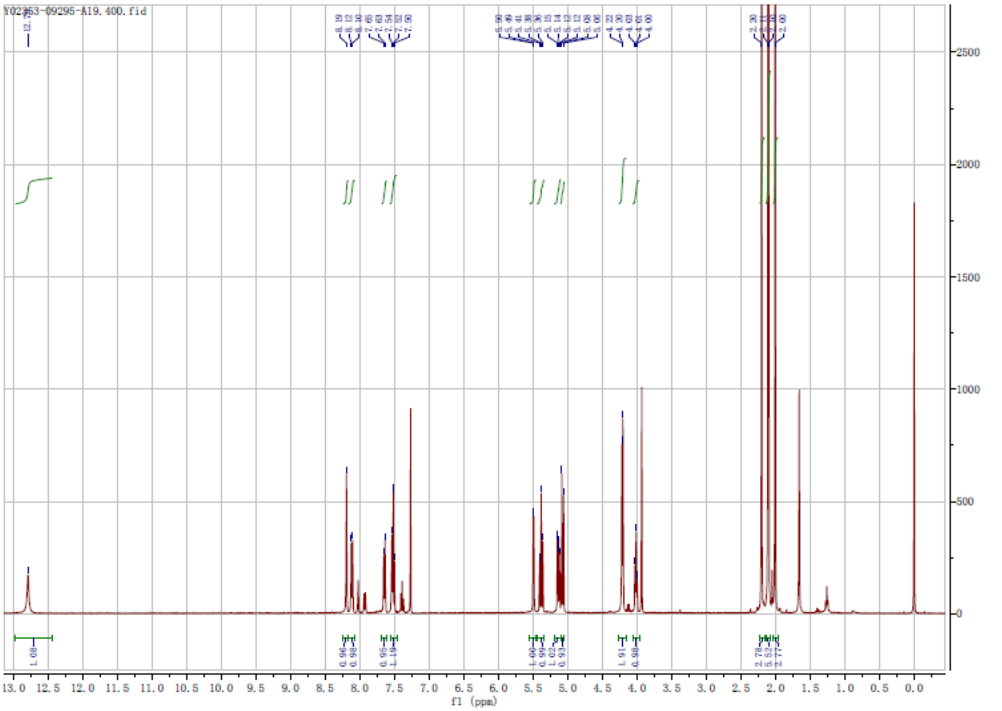

^{1}H NMR spectrum of the target compounds **III9**

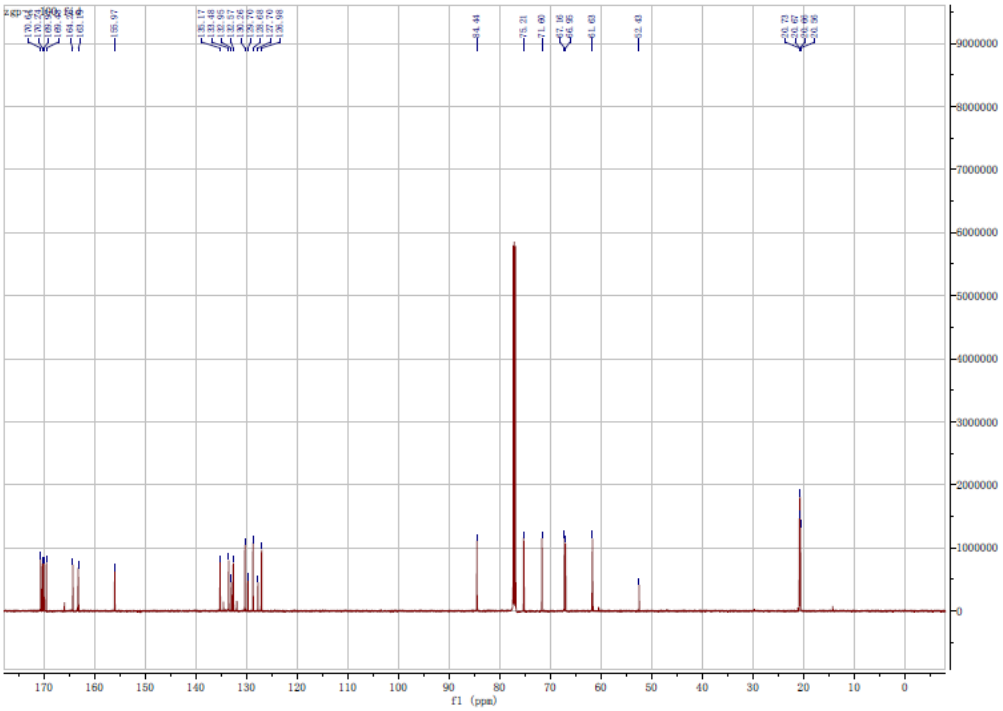

^{13}C NMR spectrum of the target compounds **III9**

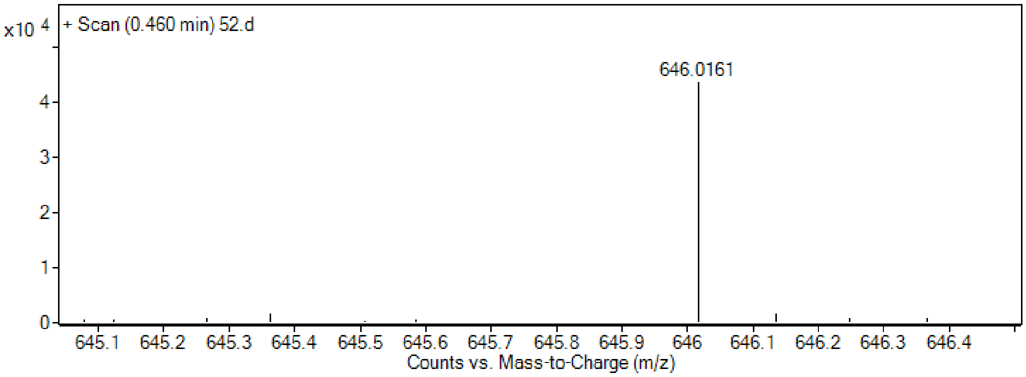

HRMS spectrum of the target compounds **III9**

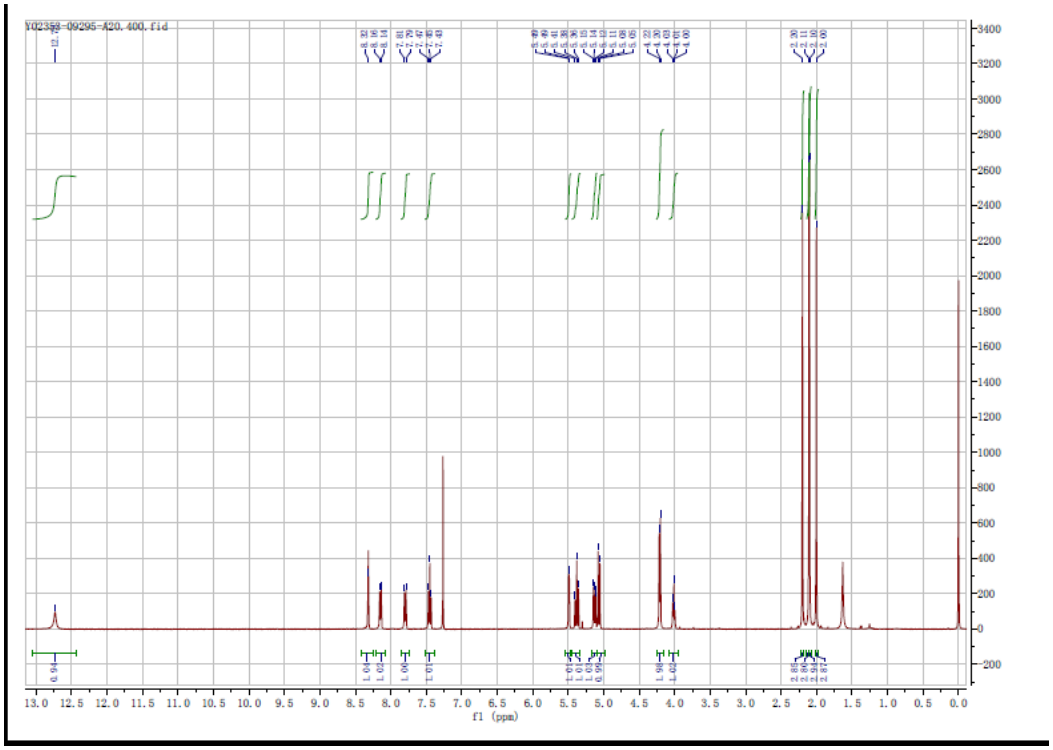

^{1}H NMR spectrum of the target compounds **III10**

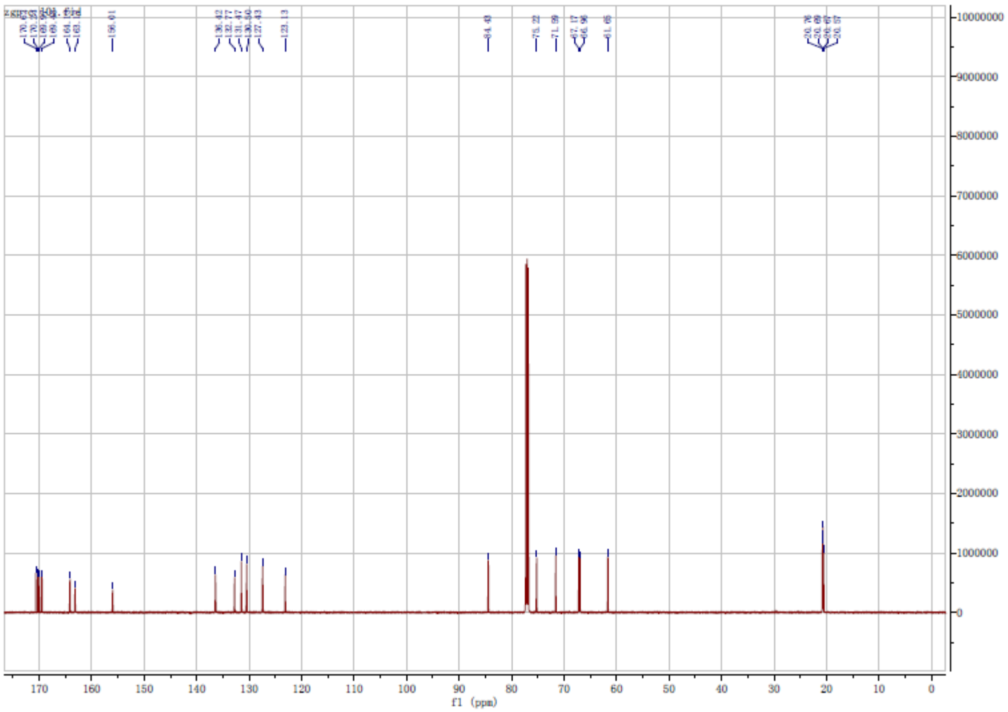

^{13}C NMR spectrum of the target compounds **III10**

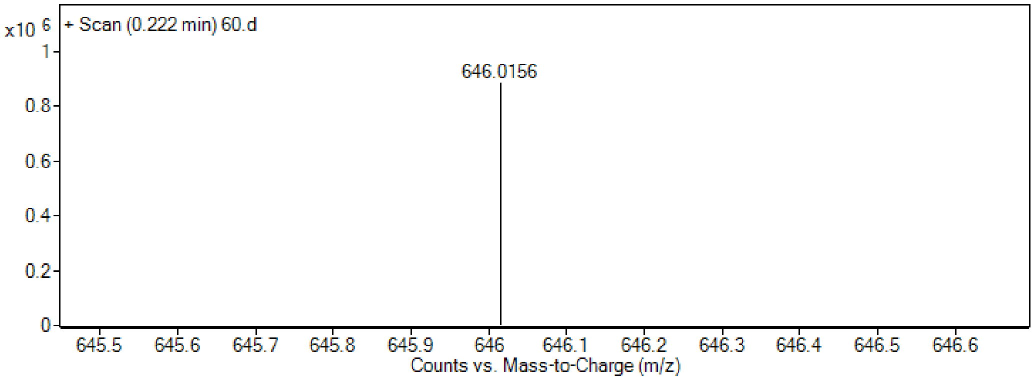

HRMS spectrum of the target compounds **III10**

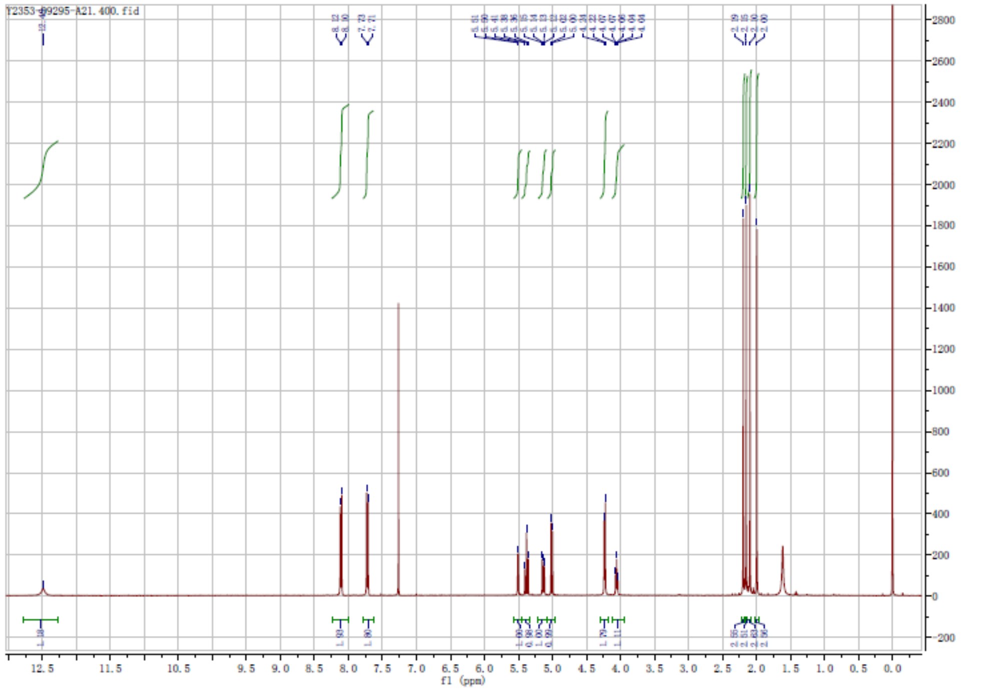

^{1}H NMR spectrum of the target compounds **III11**

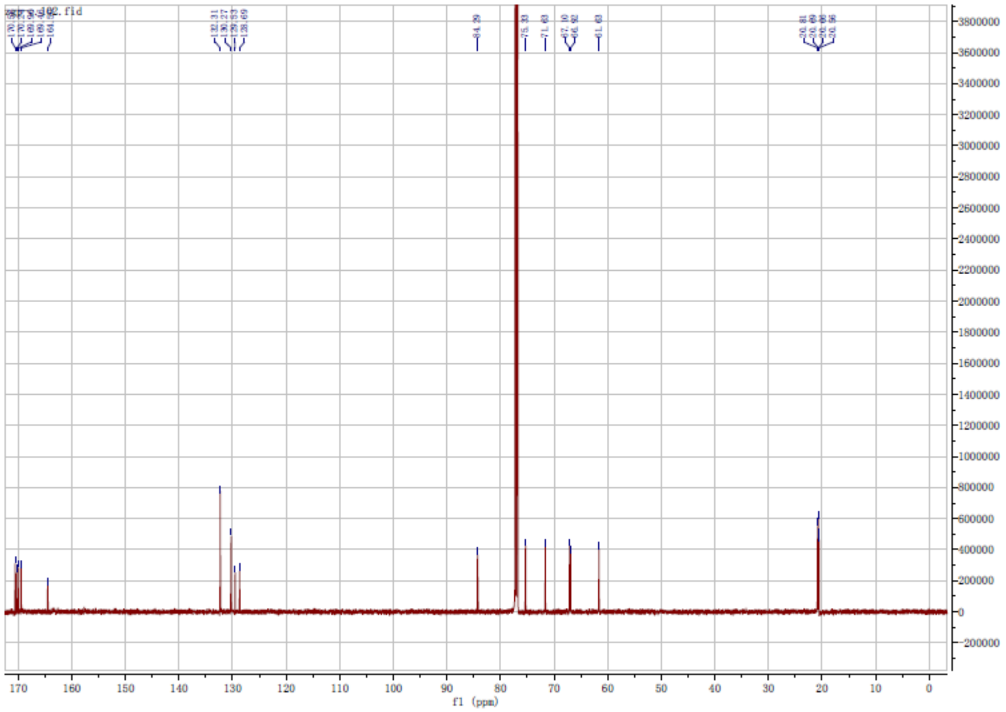

^{13}C NMR spectrum of the target compounds **III11**

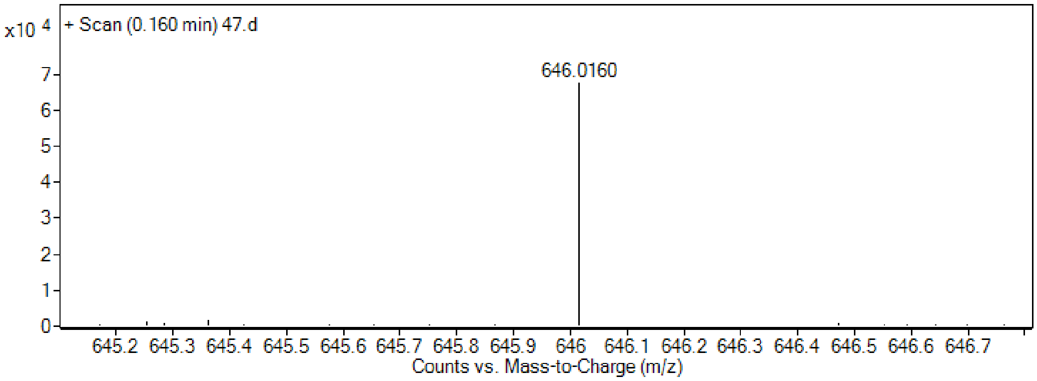

HRMS spectrum of the target compounds **III11**

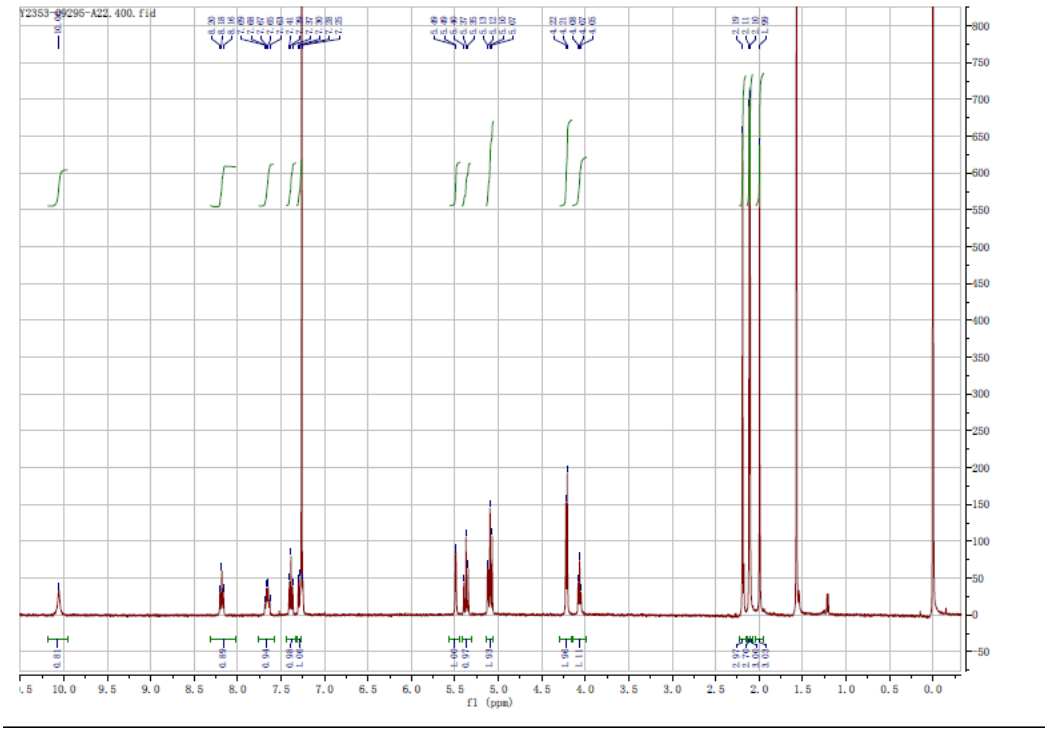

^{1}H NMR spectrum of the target compounds **III12**

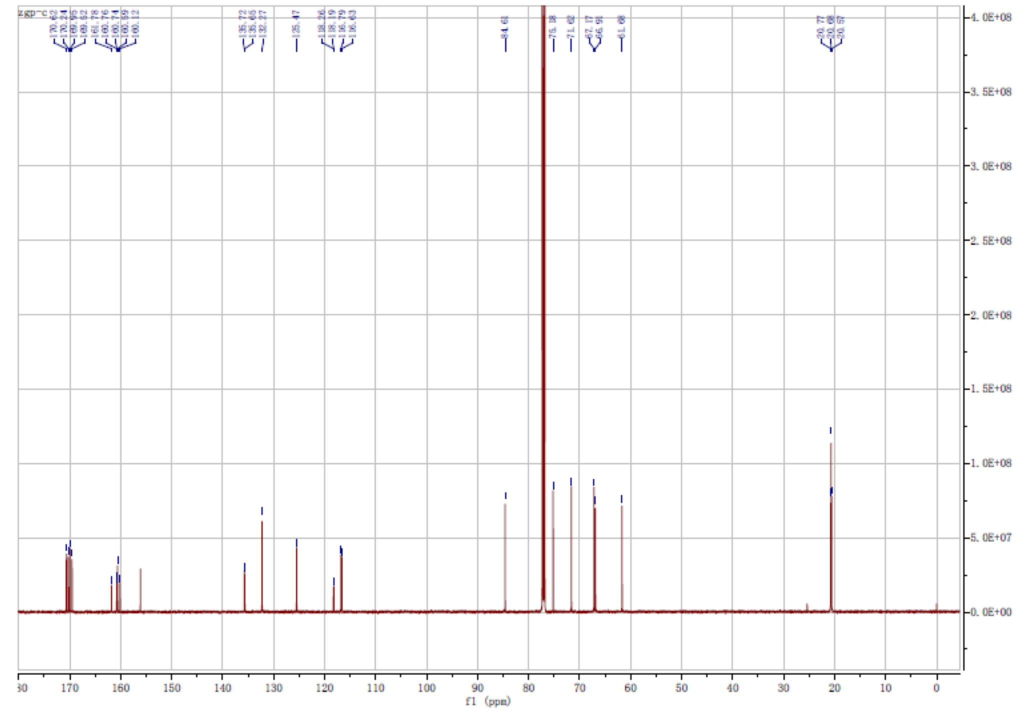

^{13}C NMR spectrum of the target compounds **III12**

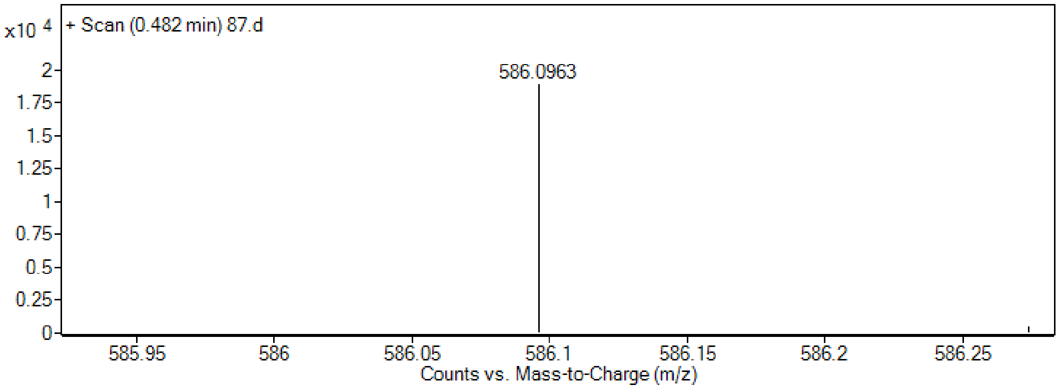

HRMS spectrum of the target compounds **III12**

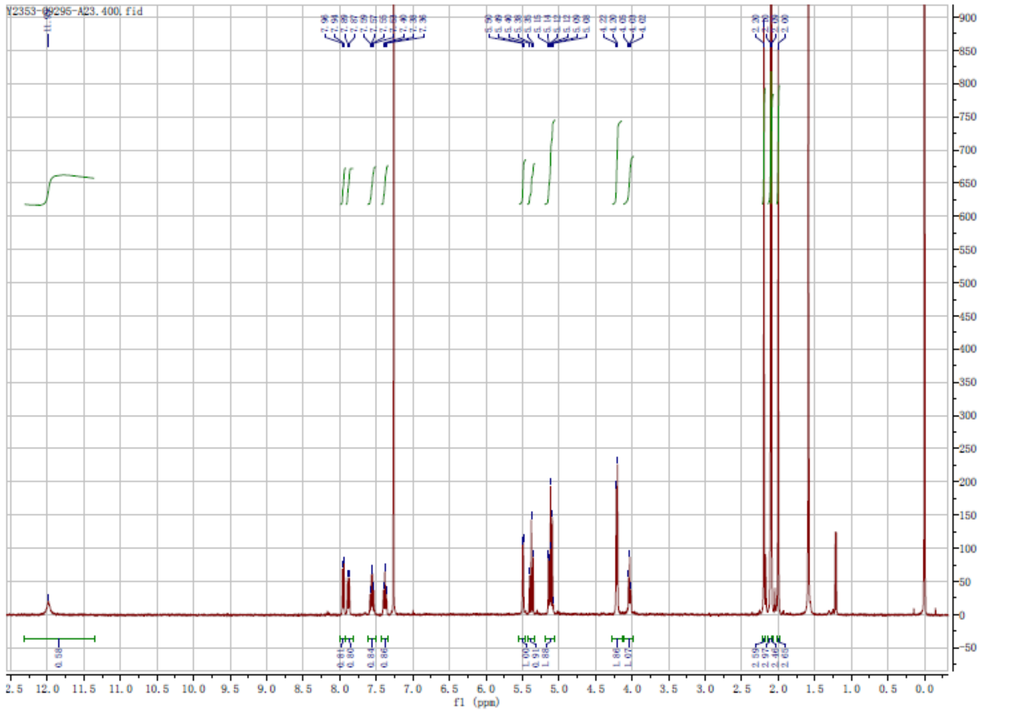

^{1}H NMR spectrum of the target compounds **III13**

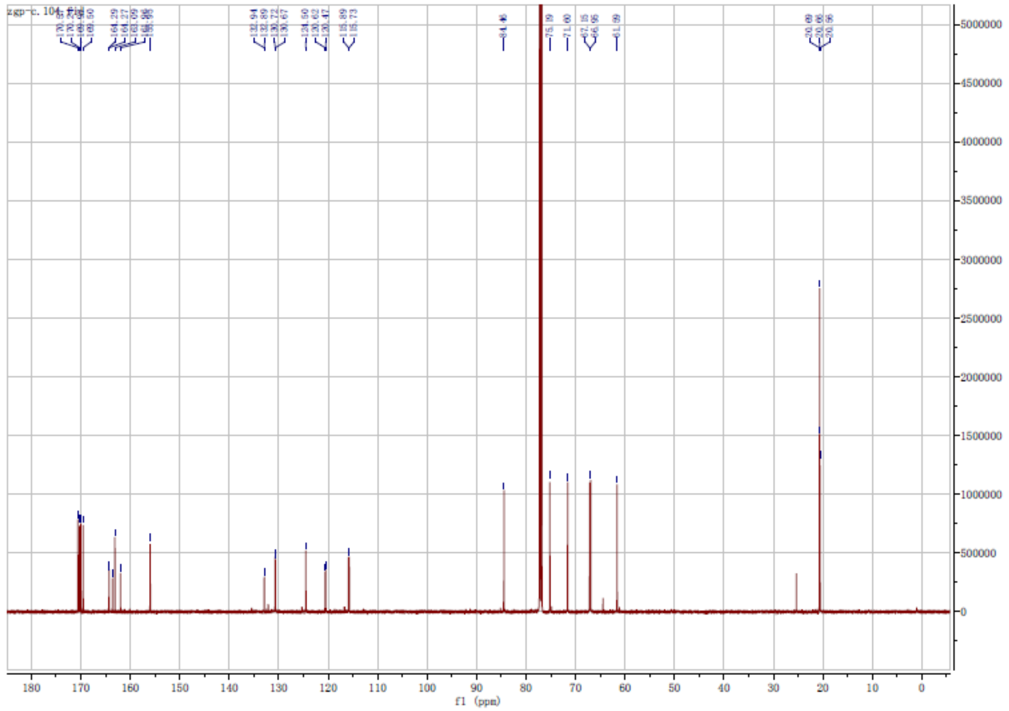

^{13}C NMR spectrum of the target compounds **III13**

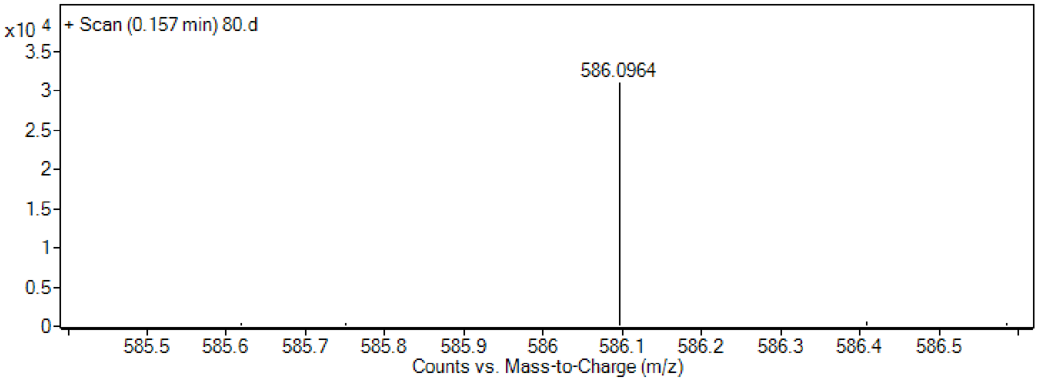

HRMS spectrum of the target compounds **III13**

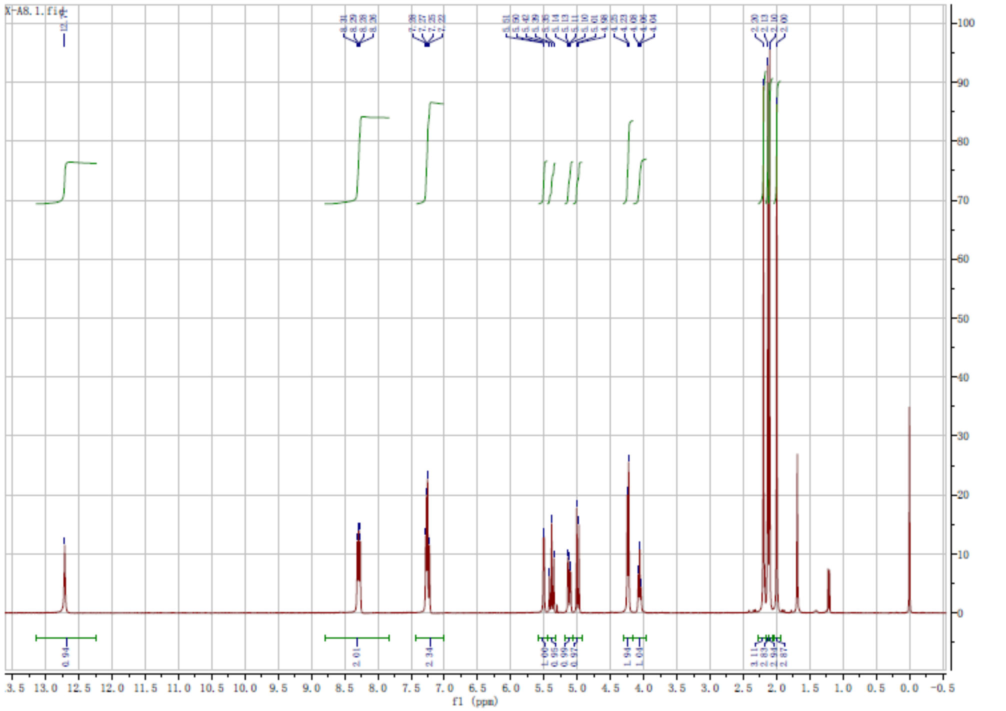

^{1}H NMR spectrum of the target compounds **III14**

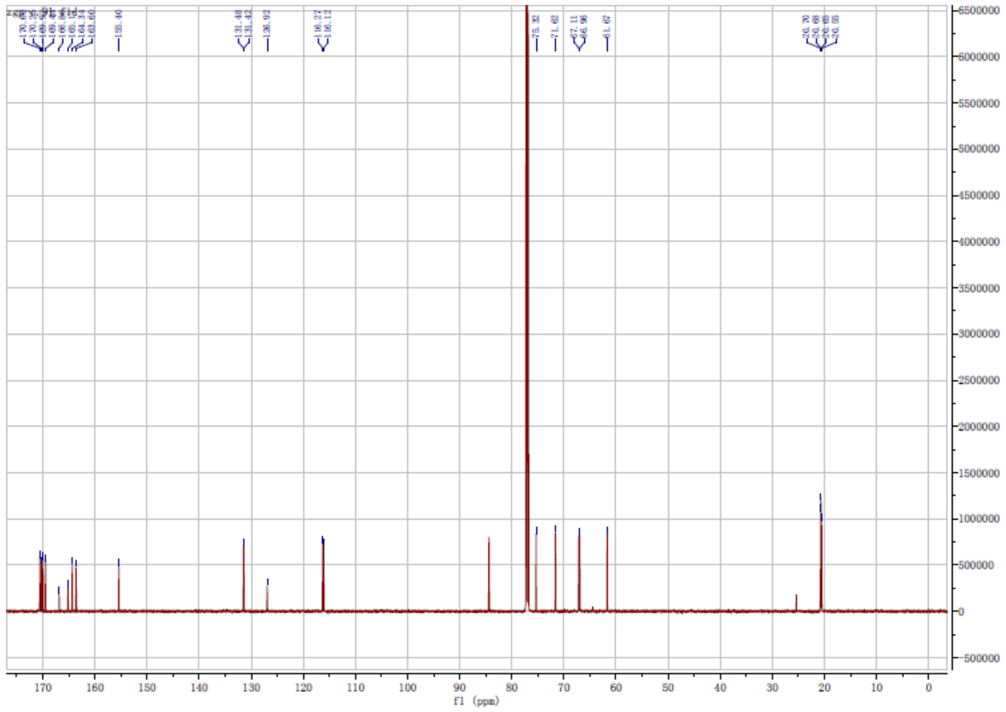

^{13}C NMR spectrum of the target compounds **III14**

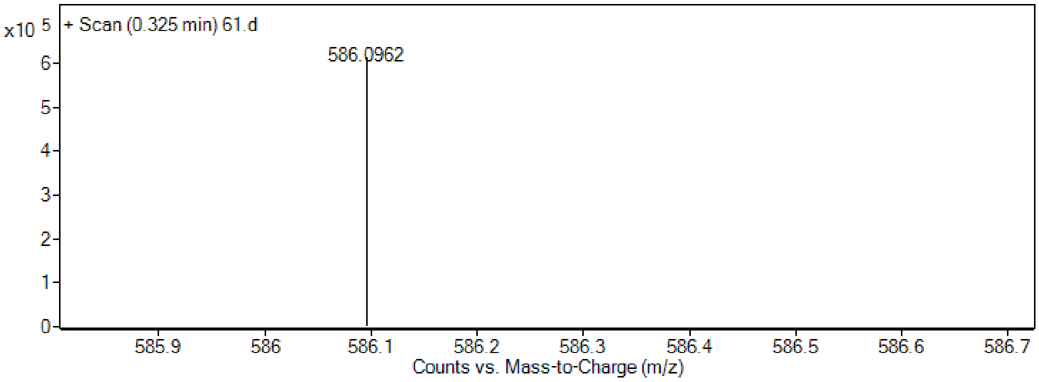

HRMS spectrum of the target compounds **III14**

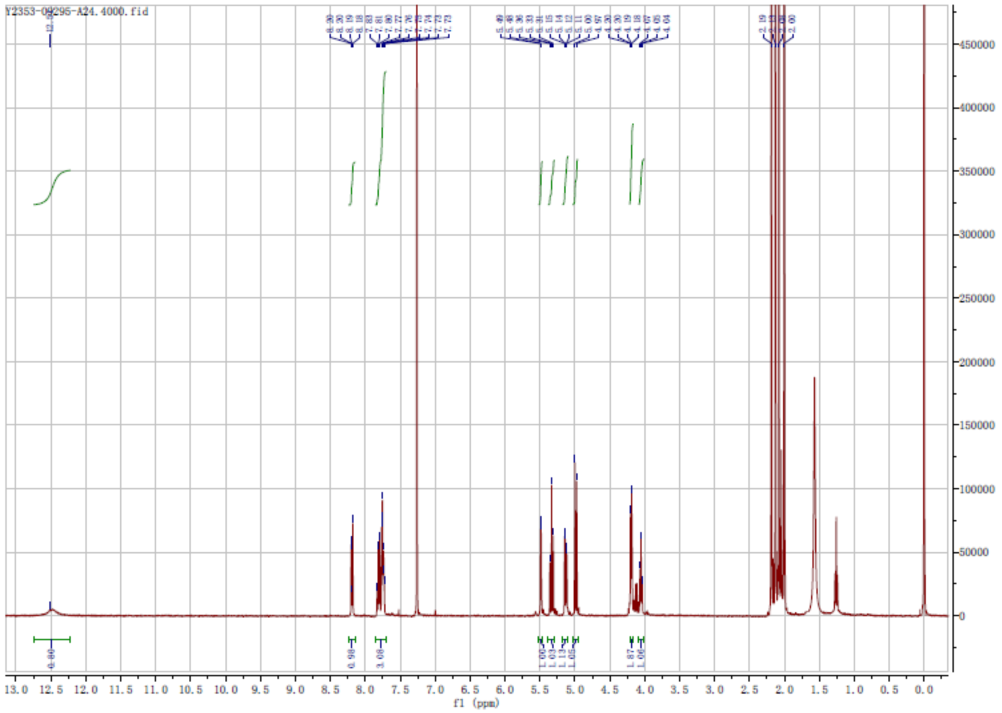

^{1}H NMR spectrum of the target compounds **III15**

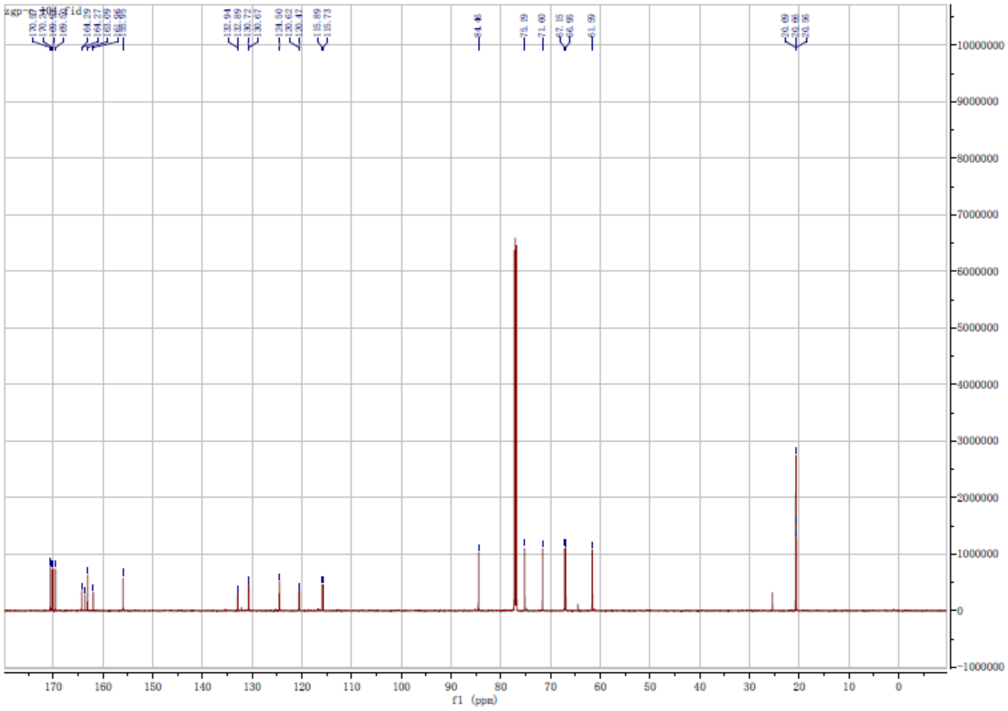

^{13}C NMR spectrum of the target compounds **III15**

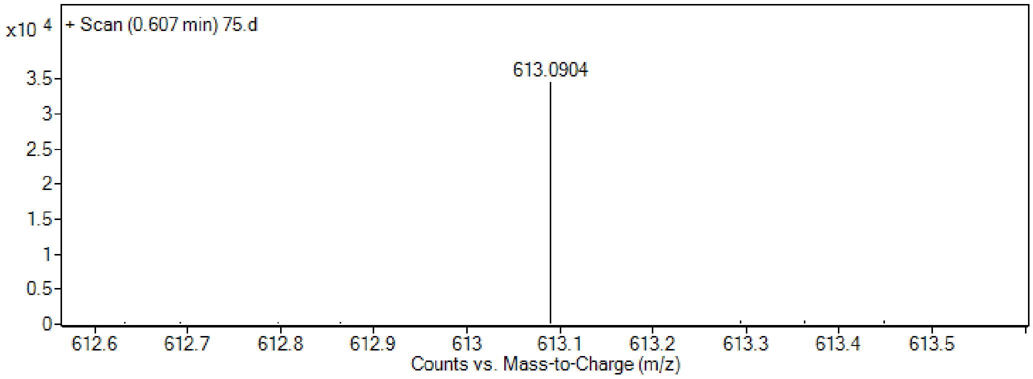

HRMS spectrum of the target compounds **III15**

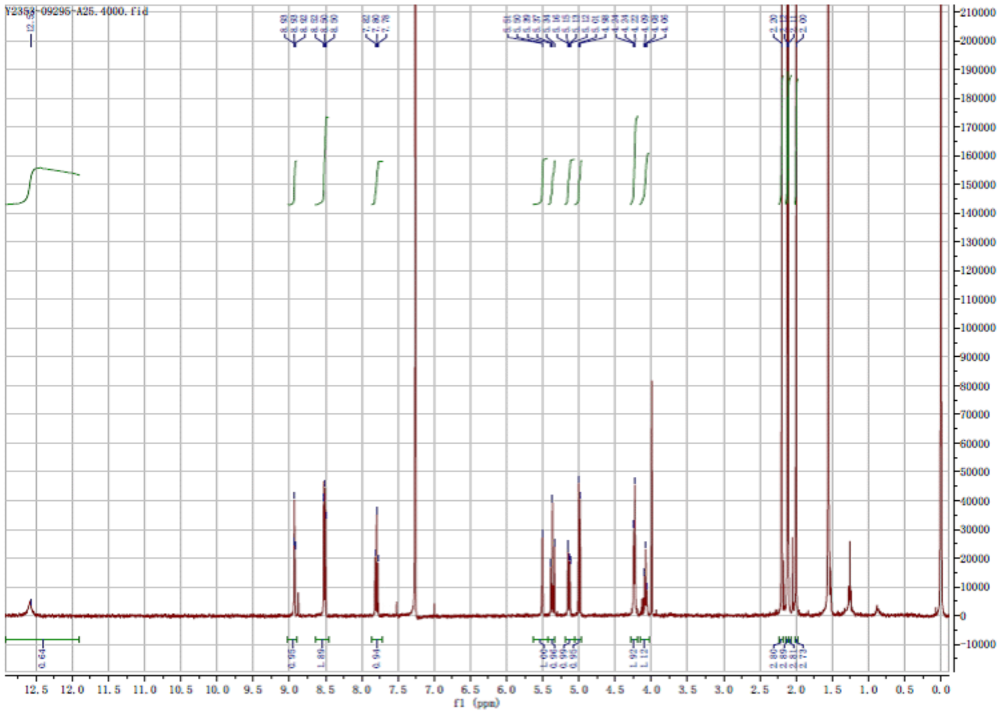

^{1}H NMR spectrum of the target compounds **III16**

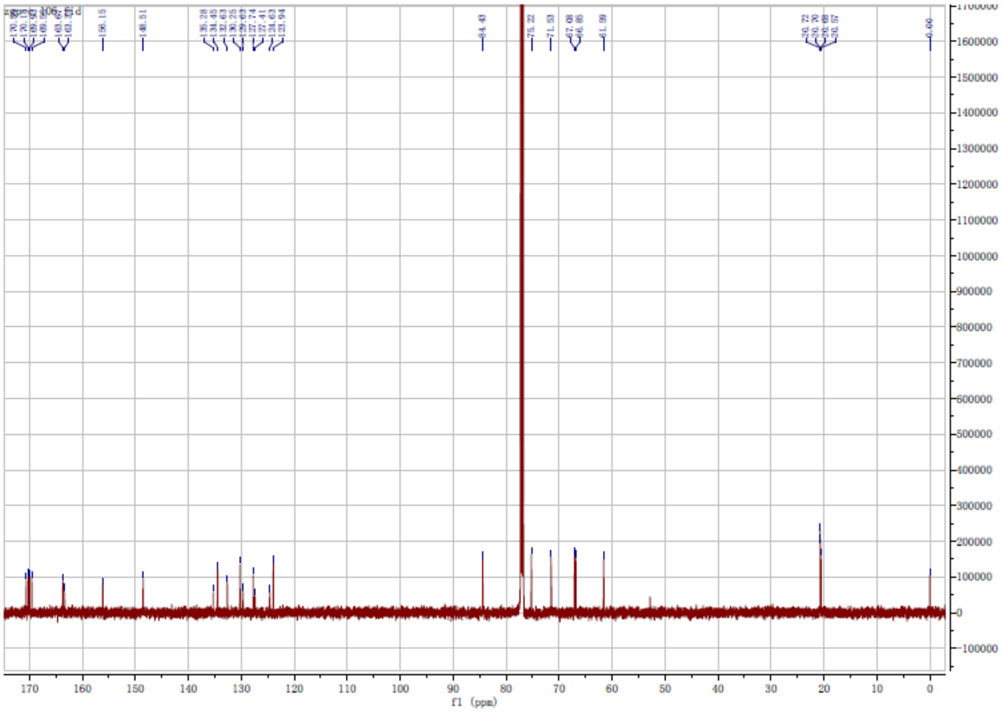

^{13}C NMR spectrum of the target compounds **III16**

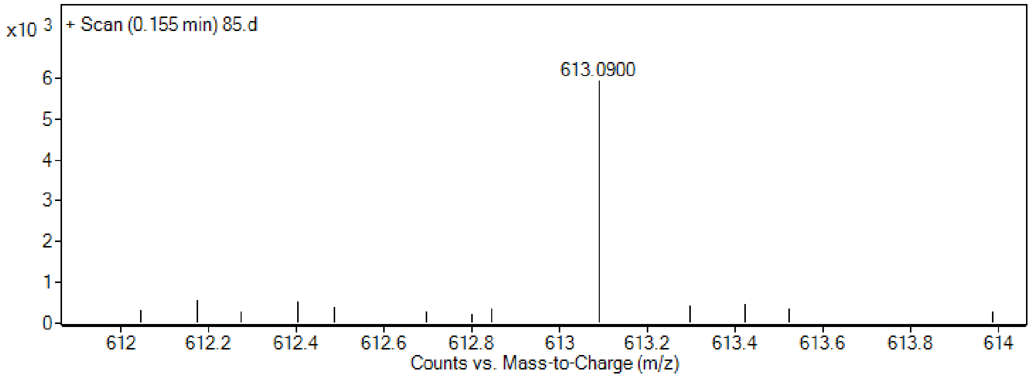

HRMS spectrum of the target compounds **III16**

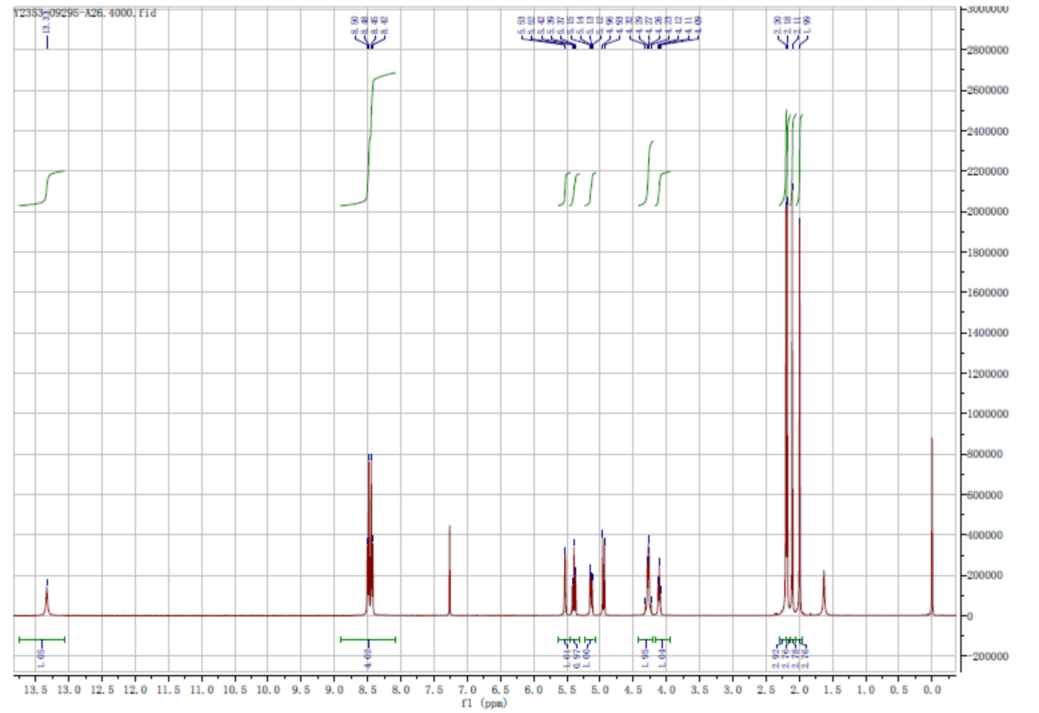

^{1}H NMR spectrum of the target compounds **III17**

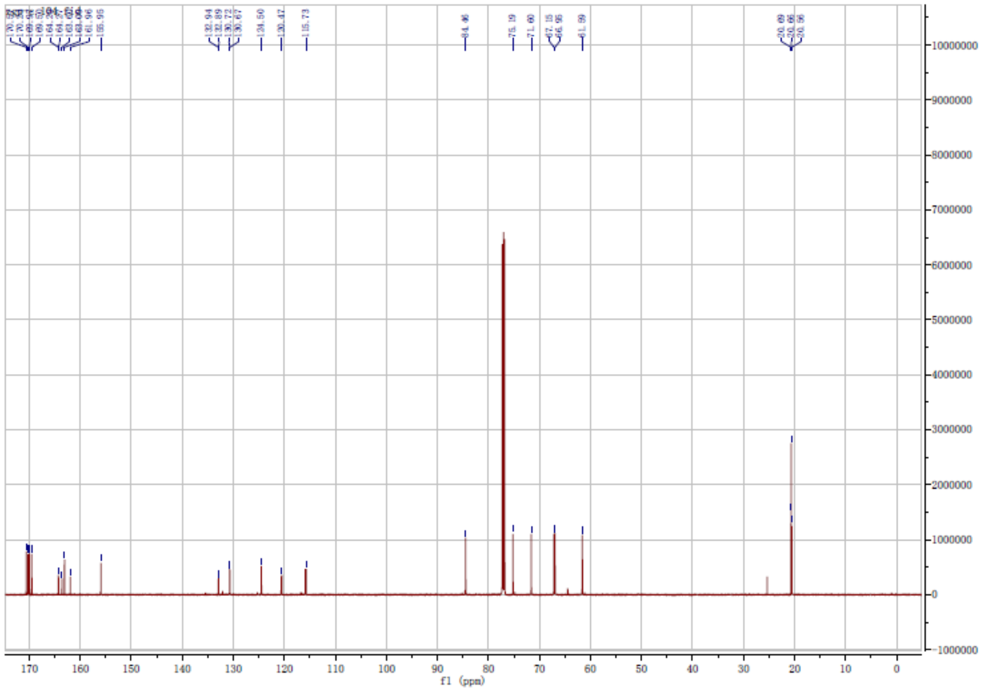

^{13}C NMR spectrum of the target compounds **III17**

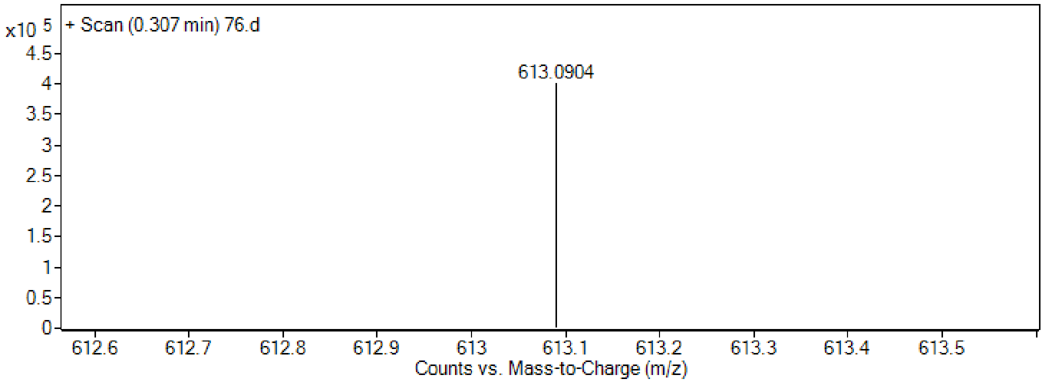

HRMS spectrum of the target compounds **III17**

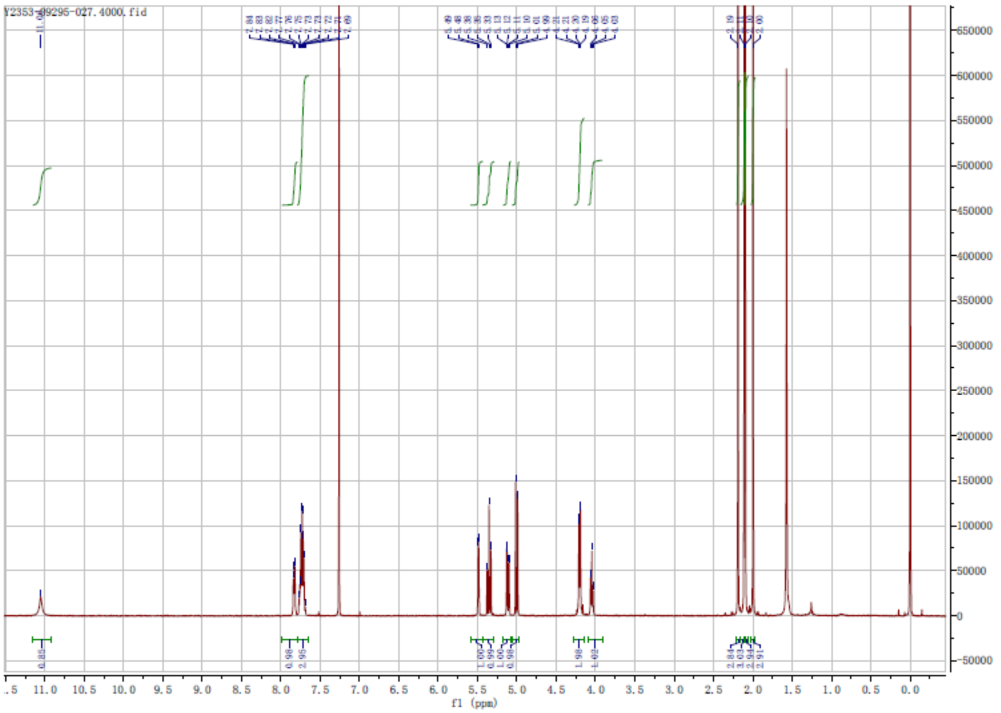

^{1}H NMR spectrum of the target compounds **III18**

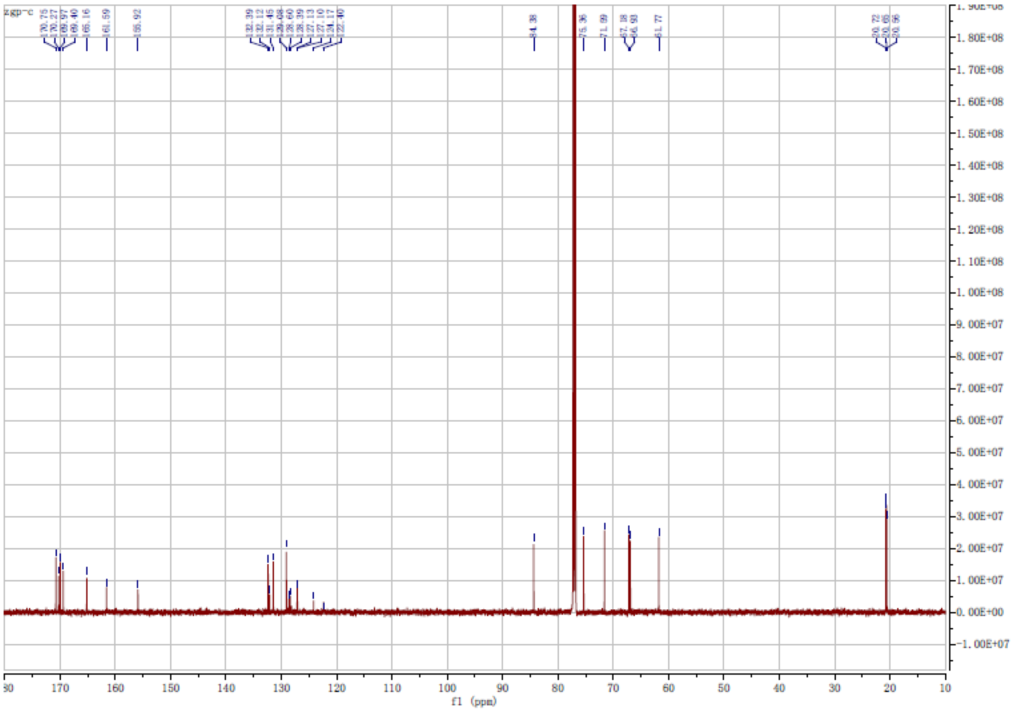

^{13}C NMR spectrum of the target compounds **III18**

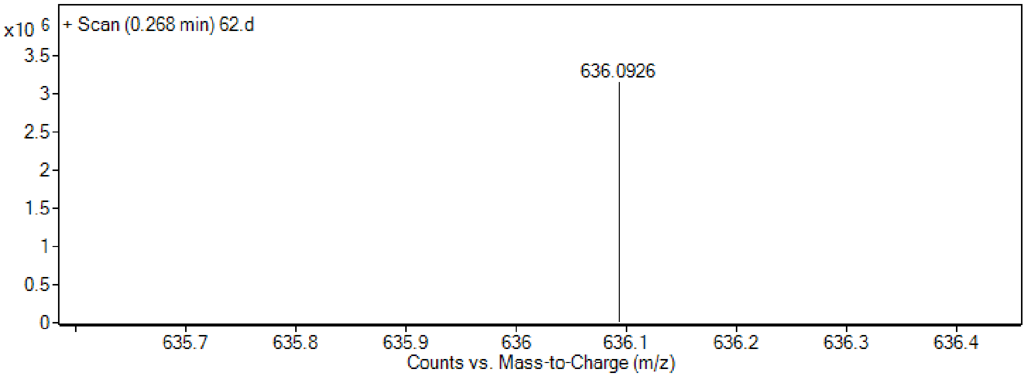

HRMS spectrum of the target compounds **III18**

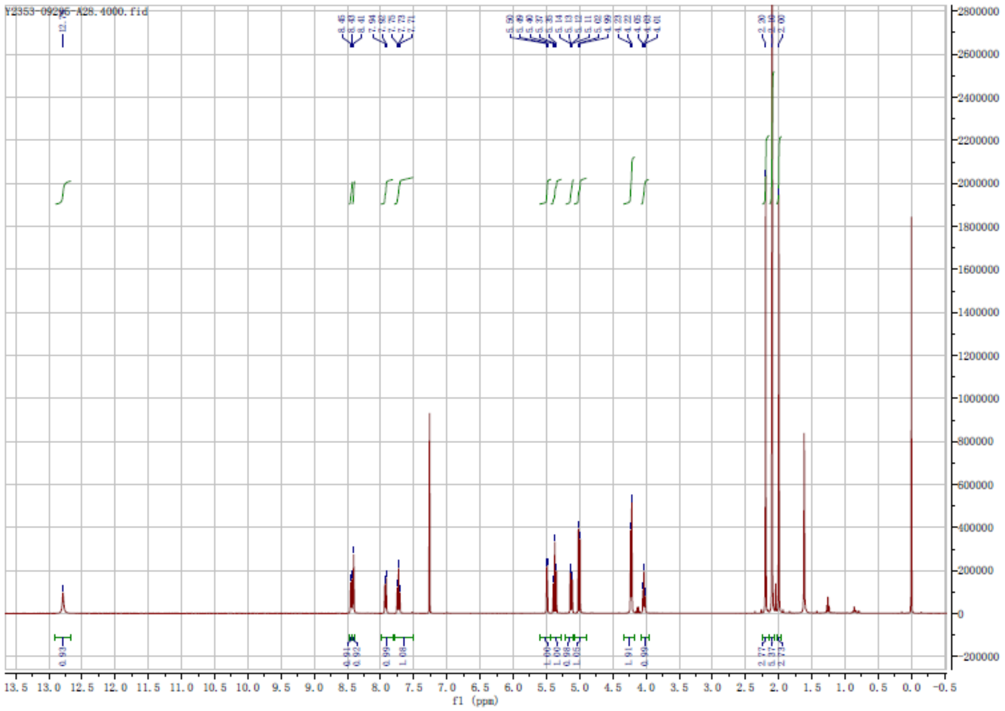

^{1}H NMR spectrum of the target compounds **III19**

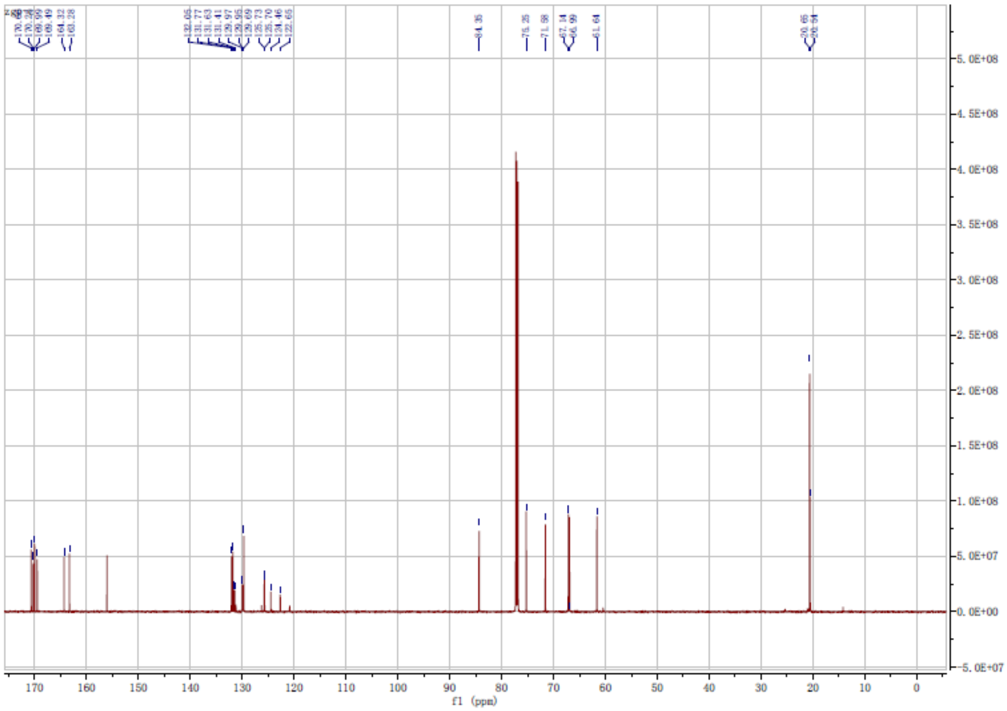

^{13}C NMR spectrum of the target compounds **III19**

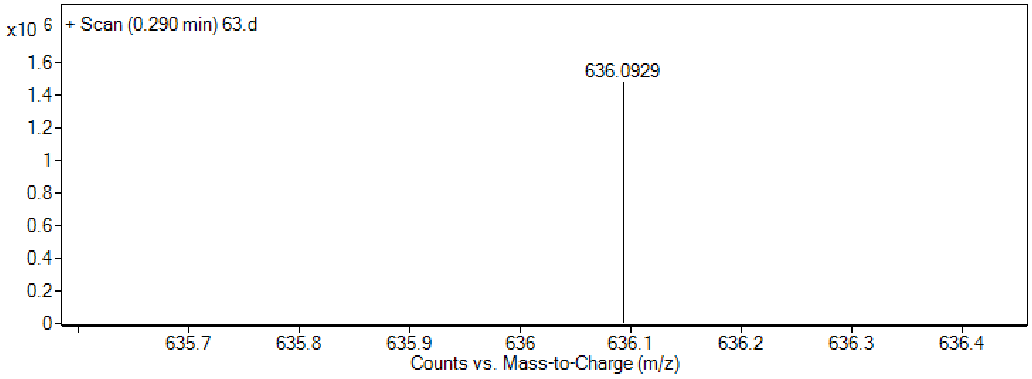

HRMS spectrum of the target compounds **III19**
